# Supplementary material for: Effectiveness, acceptability, and completeness and quality of intervention reporting of psychological interventions for people with dementia or mild cognitive impairment: protocol for a mixed-methods systematic review
Source: BMJ Open. 2023 Dec 12;13(12):e077180. doi: 10.1136/bmjopen-2023-077180 (PMC10729069; doi:10.1136/bmjopen-2023-077180)
Supplement: Supplementary data [file bmjopen-2023-077180supp002.pdf]

Database: *Ovid MEDLINE*

Search nr	Term	Comment
Concept 1 – Population		
1	exp Dementia/<br>OR exp Cognition Disorders/<br>OR Cognitive Dysfunction/	[MeSH]
2	(dement* or Alzheimer* or lewy* or frontotemporal or FTD or FTLD or tvFTD or OBS or OBD or (lewy* adj2 bod*) or organic brain syndrome* or organic brain disease or organic brain disorder* or (ADRD or AAMI or AACD or MCI or A-MCI or N-MCI or M-MCI or aMCI or MCIa or CIND or MCD or MNC or MNCD or NCD) or ag* associated cogniti* decline or ag* associated memory impairment or mild cognitive impairment or neurocognitive disorder* or preclinical AD or pre-clinical AD or (pre adj clinical AD)).ti,ab.	[Title/Abstract]
3	1 OR 2	
Concept 2 – Intervention		
4	exp Counseling/<br>OR exp Behavior Therapy/<br>OR exp Psychotherapy/<br>OR exp Bibliotherapy/	[MeSH]
5	((non adj pharmacologic*) or non-pharmacologic* or nonpharmacologic* or cognitive restructuring or cognitive reframing or behavio* activation or activity scheduling or problem solving or (cCBT or iCBT or ehealth or e-health or teletherapy or telehealth) or (self adj help) or (self adj manag*) or (self adj administer*) or (psycho* adj therapy) or (cognitive adj2 therap*) or (behavio* adj2 therap*) or (CBT or psychotherapy or psychodynamic or counseling or counselling or psychoeducation* or psychosocial or psycho-social) or (group adj therap*) or (group adj treatment*) or (group adj intervention*) or (group adj support) or psycho education*).ti,ab.	[Title/Abstract]
6	4 OR 5	
Concept 3 – Outcome		
7	exp Depression/<br>OR exp Anxiety/<br>OR exp Anxiety Disorders/<br>OR exp Mood Disorders/<br>OR Affective Symptoms/<br>OR Psychological Distress/	[MeSH]
8	("quality of life" or (mental adj health) or (mental adj distress) or (mental adj illness) or (psycholo*	[Title/Abstract]

	adj distress) or (neuropsycholog* or neuropsychiatric) or (mood or emotion* or affective or wellbeing or well-being or distress) or (negative adj affect) or Depress* or Melanchol* or Dysphori* or (anxiety or anxious or stress or worr*) or (well adj being)).ti,ab.	
9	7 OR 8	
Concept 4 – Study design		
10	exp Randomized Controlled Trial/<br>OR exp Clinical Trial/<br>OR Meta-Analysis/<br>OR Random Allocation/	[MeSH]
11	(random* controlled trial* or (RCT or Trial or review or meta-analysis) or (random* adj allocat*)).ti,ab.	[Title/Abstract]
12	10 OR 11	
Final results	3 AND 6 AND 9 AND 12	
