# Supplementary material for: Effectiveness, acceptability, and completeness and quality of intervention reporting of psychological interventions for people with dementia or mild cognitive impairment: protocol for a mixed-methods systematic review
Source: BMJ Open. 2023 Dec 12;13(12):e077180. doi: 10.1136/bmjopen-2023-077180 (PMC10729069; doi:10.1136/bmjopen-2023-077180)
Supplement: Author's manuscript [file bmjopen-2023-077180.draft_revisions.pdf]

BMJ Open is committed to open peer review. As part of this commitment we make the peer review history of every article we publish publicly available.

When an article is published we post the peer reviewers' comments and the authors' responses online. We also post the versions of the paper that were used during peer review. These are the versions that the peer review comments apply to.

The versions of the paper that follow are the versions that were submitted during the peer review process. They are not the versions of record or the final published versions. They should not be cited or distributed as the published version of this manuscript.

BMJ Open is an open access journal and the full, final, typeset and author-corrected version of record of the manuscript is available on our site with no access controls, subscription charges or pay-per-view fees (http://bmjopen.bmj.com).

If you have any questions on BMJ Open's open peer review process please email info.bmjopen@bmj.com

Effectiveness, acceptability, and completeness and quality of intervention reporting of psychological interventions for people with dementia or mild cognitive impairment: protocol for a mixed-methods systematic review

Journal:	*BMJ Open*
Manuscript ID	bmjopen-2023-077180
Article Type:	Protocol
Date Submitted by the Author:	27-Jun-2023

Complete List of Authors:	Svedin, Frida; Uppsala University, Women's and Children's Health<br>Blomberg, Oscar; Uppsala University, Department of Women's and Children's Health<br>Farrand, Paul; University of Exeter, Clinical Education Development and Research (CEDAR)<br>Brantnell, Anders; Uppsala University, Department of Women's and Children's Health; Uppsala University, Department of Civil and Industrial Engineering<br>von Essen, Louise; Uppsala University, Department of Women's and Children's Health<br>Åberg, Anna ; Uppsala University, Department of Public Health and Caring Sciences; Dalarna University, School of Health and Welfare<br>Woodford, Joanne; Uppsala University, Department of Women's and Children's Health

Keywords:	Dementia < NEUROLOGY, MENTAL HEALTH, PSYCHIATRY, Depression & mood disorders < PSYCHIATRY, Anxiety disorders < PSYCHIATRY, Old age psychiatry < PSYCHIATRY

SCHOLARONE™
Manuscripts

Effectiveness, acceptability, and completeness and quality of intervention reporting of psychological interventions for people with dementia or mild cognitive impairment: protocol for a mixed-methods systematic review

Frida SVEDIN[1] (frida.svedin@kbh.uu.se), Oscar BLOMBERG[1] (oscar.blomberg@kbh.uu.se), Paul FARRAND[3] (p.a.farrand@exeter.ac.uk), Anders BRANTNELL[1,2] (anders.brantnell@kbh.uu.se), Louise von ESSEN[1] (louise-von.essen@kbh.uu.se), Anna Cristina ÅBERG[4,5] (anna.cristina.aberg@pubcare.uu.se), Joanne WOODFORD[1] (joanne.woodford@kbh.uu.se)

Corresponding author

Dr Joanne Woodford, Healthcare Sciences and e-health, Department of Women's and Children's Health, Uppsala University, 751 85 Uppsala, Sweden. E-mail: joanne.woodford@kbh.uu.se. Telephone: +4618 471 35 64

Affiliations

[1]Healthcare Sciences and e-Health, Department of Women's and Children's Health, Uppsala University, 751 85 Uppsala, Sweden.

[2]Industrial Engineering and Management, Department of Civil and Industrial Engineering, Uppsala University, 751 21 Uppsala, Sweden.

[3]Clinical Education, Development and Research (CEDAR); Psychology, University of Exeter, Perry Road, Devon, EX4 4QG, United Kingdom.

[4]Clinical Geriatrics, Department of Public Health and Caring Sciences, Uppsala University, 751 85 Uppsala, Sweden.

[5]Medical Science, School of Health and Welfare, Dalarna University, 791 88 Falun, Sweden.

For peer review only

ABSTRACT

Introduction: Mental health difficulties such as anxiety and depression have negative impacts on psychological well-being, and are common in people with dementia and mild cognitive impairment. However, access to psychological treatments is limited. This mixed-method systematic review will: (1) examine the effectiveness of psychological interventions to improve mental health and psychological well-being in people with dementia or mild cognitive impairment; (2) examine the effectiveness of these psychological interventions to improve mental health and psychological well-being in informal caregivers; (3) examine potential clinical and methodological moderators associated with effectiveness; (4) explore factors associated with the acceptability of psychological interventions from the perspective of key stakeholders; and (5) examine the completeness and quality of intervention reporting.

Methods and analysis: Electronic databases (ASSIA, CENTRAL, CINAHL; EMBASE; PsychInfo; and MEDLINE) will be systematically searched and supplemented with expert contact, reference and citation checking, and grey literature searches. If possible, we will conduct a meta-analysis to examine the overall effectiveness of psychological interventions to improve mental health and psychological well-being in people with dementia or mild cognitive impairment and their informal caregivers; and examine potential clinical and methodological moderators associated with effectiveness. We will conduct a deductive framework synthesis, informed by the Theoretical Framework of Acceptability, to explore factors associated with the acceptability of psychological interventions from the perspective of key stakeholders. In accordance with JBI guidance, we will adopt a convergent segregated approach to data synthesis and integration of quantitative and qualitative findings. We will examine the completeness and quality of intervention reporting according to the Template for Intervention Description and Replication (TIDieR) checklist and guide.

Ethics and dissemination: No primary data will be collected and therefore ethical approval is not required. Results will be disseminated through a peer-reviewed publication, academic conferences, and plain language summaries.

PROSPERO registration number: CRD42023400514.

Key Words: Dementia; MCI; Psychological Well-being; Depression; Anxiety; Informal caregivers; Mixed-methods Review

For peer review only - http://bmjopen.bmj.com/site/about/guidelines.xhtml

Strengths and limitations of this study:

- This will review will extend the existing literature by including novel aspects such as narrowing the classification and definition of eligible psychological interventions, excluding studies with a high risk of bias, and exploring intervention acceptability and the completeness of intervention reporting.
- We will adopt a mixed-method approach informed by the convergent segregated approach, allowing us to synthesize evidence from multiple sources to address review questions.
- This review will follow quality standards informed by the New Cochrane Handbook for Systematic Reviews of Interventions, PRISMA 2020 guidelines, and Enhancing transparency in reporting the synthesis of qualitative research (ENTREQ).
- Despite the Critical Appraisal Skills Programme (CASP) checklist being a recommended tool for assessing the quality of qualitative studies, it does not include a score rating system for scoring the quality of qualitative studies. This may lead to inclusion of low-quality qualitative studies.
- Due to limited resources, only studies available in English and Swedish language will be included.

For peer review only - http://bmjopen.bmj.com/site/about/guidelines.xhtml

INTRODUCTION

Dementia is a global public health priority,[1] with the number of people living with dementia estimated to rise from 50 million in 2016 to 152 million in 2050.[2] Dementia has several significant adverse impacts, including increased mortality,[3] cognitive [4] and functional decline,[5] and poor quality of life.[6] Significant demands are placed on informal caregivers (i.e., family and friends who provide unpaid care) and the wider society.[7] People with dementia and mild cognitive impairment (MCI) commonly experience co-morbid mental health difficulties, such as anxiety and depression,[8-11] with anxiety and depression being associated with exacerbation of cognitive decline, poor quality of life, sleep difficulties, and reduction in activities of daily living.[12] For people with dementia, the overall pooled prevalence is estimated to be 39% for both anxiety and for depression,[10] and for people with MCI 21% for depression[8] and 32% for anxiety.[9] Despite research suggesting psychological interventions are effective,[13-15] access to evidence-based psychological support for people with dementia is limited.[16, 17]

Recent reviews suggest that psychological interventions for anxiety and depression in dementia or MCI are probably effective.[13-15, 18] However, these reviews have included a broad range of psychosocial interventions (e.g., psychosocial interventions focusing on psychological and social factors, relaxation training therapies, music therapies, exercise, and massage) yielding large heterogeneity in intervention type. To facilitate subgroup analyses to explore the potential moderating effect of psychological intervention type, we intend to build upon previous reviews by using a narrower classification of psychological interventions.[19] Using a narrower classification and thus potentially reducing clinical heterogeneity may facilitate the identification of clinical moderators rarely investigated such as intervention delivery model (e.g., individual, dyadic, group, or self-administered) and method of support (e.g., face-to-face, internet, telephone, or video-conference).

Previous reviews[13-15, 18] have not examined the effectiveness of psychological interventions targeting psychological well-being in people with dementia or MCI.[20] Psychological interventions targeting psychological well-being may be of particular importance given the challenges experienced in diagnosing people with dementia and depression due to overlapping symptomology between late-life depression and dementia.[21] Diagnosis difficulties are also experienced in diagnosing anxiety, given people with dementia or MCI may present with anxiety symptoms inconsistent with standardized diagnostic

For peer review only - http://bmjopen.bmj.com/site/about/guidelines.xhtml

criteria.[22] In addition, informal caregivers are at an increased risk of experiencing difficulties with their mental health and psychological well-being, for example experiencing burden and depression,[23] and poor quality of life.[24] Given the dyadic interdependence between the care recipient and caregiver's psychological well-being,[25] it may also be important to examine whether psychological interventions to improve mental health and psychological well-being in people with dementia or MCI also improve the mental health and psychological well-being in informal caregivers. In a previous review examining the effect of psychological treatments for anxiety and depression for dementia and MCI a small number of studies were found that included informal caregiver outcomes.[14] However, how interventions aimed at improving psychological well-being or mental health-related quality of life in people with dementia and MCI may also impact informal caregiver mental health and psychological well-being warrants further investigation. Finally, existing reviews have included studies with non-randomized designs[13] or studies with a high risk of bias concerning randomization procedures.[15] Including these studies may result in an overestimation of the treatment effect.[26, 27] Consequently, there is a need to conduct a systematic review excluding studies of low methodological quality.

In addition to the aforementioned gaps in the evidence base, people with dementia or MCI commonly experience barriers to accessing psychological support. Barriers include factors related to the individual (e.g., lack of time for treatment or geographic distance), the provider (e.g., lack of willingness to diagnose and treat mental health difficulties), and the healthcare system (e.g., limited availability of specialty mental health providers).[28] Barriers commonly experienced by people with dementia or MCI include social stigmatization at the individual level,[29] and lack of knowledge of mental health problems among healthcare providers[17, 30] at the provider level. An additional barrier to access is intervention acceptability, i.e. the anticipated or experienced cognitive and emotional response to an intervention from the perspective of those receiving and delivering the intervention.[31] For example, lack of motivation and low patient interest represent common barriers to intervention access among older adults.[32] It is essential to consider acceptability when developing, evaluating, and implementing healthcare interventions[31, 33] given associations with intervention adherence[31] and positive clinical outcomes.[34] However, to date, little is known about the acceptability of psychological interventions for people with dementia or MCI, and other important stakeholders (e.g., informal caregivers and healthcare professionals). Considering factors associated with acceptability[35] may help improve treatment access and enhance the

For peer review only - http://bmjopen.bmj.com/site/about/guidelines.xhtml

implementation potential of future developed interventions by increasing intervention acceptance and relevance from the perspective of those receiving and delivering/implementing the intervention.

An additional barrier to treatment access may relate to the completeness and quality of the reporting of psychological interventions in the research literature. The completeness and quality of published intervention descriptions (i.e., descriptions of evaluated interventions that are detailed enough to facilitate replication) are commonly poor.[36, 37] Consequently, reliable replication and/or implementation of interventions is difficult.[36] Ensuring the transparency and completeness of intervention reporting can facilitate the adoption of interventions by clinicians, informal caregivers, patients, and policy decision-makers,[38] and is essential for implementing interventions in practice.[39] Existing reviews suggest intervention components of psychological interventions for people with dementia or MCI are poorly reported.[14] However, to the best of our knowledge, there is no comprehensive review describing the quality and completeness of intervention reporting of psychological interventions for people with dementia or MCI.

In summary, this mixed-methods systematic review will address the aforementioned gaps in the literature by: (1) using a narrower and commonly used classification of psychological interventions,[19] to reduce clinical heterogeneity and facilitate comparison of findings and exploration of potential clinical moderators; (2) examining the effectiveness of psychological interventions for people with dementia or MCI on general psychological well-being and mental health-related quality of life; (3) examining the effectiveness of these psychological interventions to improve the mental health and psychological well-being in informal caregivers; (4) excluding studies with non-randomized and uncontrolled designs and RCTs with randomization procedures providing a high risk of bias, in line with the Cochrane Collaboration's Risk of Bias tool 2.0 (RoB 2.0);[40] (5) exploring intervention acceptability from the perspective of important stakeholders (e.g., people with dementia or MCI, informal caregivers, and healthcare professionals); and (6) examining intervention reporting quality and completeness.

Objectives

This mixed-method systematic review will: (1) examine the effectiveness of psychological interventions to improve mental health and psychological well-being in people with dementia

For peer review only - http://bmjopen.bmj.com/site/about/guidelines.xhtml

or mild cognitive impairment; (2) examine the effectiveness of these psychological interventions to improve mental health and psychological well-being in informal caregivers; (3) examine potential clinical and methodological moderators associated with effectiveness; (4) explore factors associated with the acceptability of psychological interventions from the perspective of key stakeholders; and (5) examine the completeness and quality of intervention reporting.

METHODS AND ANALYSIS

The review will adopt a mixed methods approach with the protocol reported in accordance with the Preferred Reporting Items for Systematic Reviews and Meta-Analyses Protocols (PRISMA-P) statement[41] and Enhancing Transparency in Reporting the Synthesis of Qualitative Research (ENTERQ) statement.[42] Study results will be reported following the Preferred Reporting Items for Systematic Reviews and Meta-Analyses 2020 (PRISMA 2020) guidelines.[43] This protocol has been registered with the International Prospective Register of Systematic Reviews (PROSPERO) registration number: CRD42023400514 and is partially informed by a previous review protocol.[44] Centre of Reviews and Dissemination (CRD) guidance for conducting systematic reviews,[45] the New Cochrane Handbook for Systematic Reviews of Interventions,[46] and JBI guideance for mixed methods systeamtic reviews will be followed.[47]

Inclusion and exclusion criteria

Participants

Adults diagnosed with dementia or MCI in accordance with a validated diagnostic criterion (e.g., the Diagnostic and Statistical Manual (DSM), International Classification of Diseases (ICD) other validated criteria), or recorded in medical records. Eligible dementia diagnoses include Alzheimer’s disease and related dementias (e.g., cerebrovascular/vascular disease, Frontotemporal dementia, Lewy body disease, or mixed). Adults diagnosed with dementia primarily due to human immunodeficiency virus, Huntington's disease, Parkinson's disease, Prion disease, substance/medication use, traumatic brain injury, multiple sclerosis, or other medical conditions will be excluded. No restrictions will be placed on dementia severity, or time since diagnosis. Community and institutional-dwelling persons with dementia or MCI will be eligible for inclusion. Persons with comorbid severe mental health problems (e.g., bipolar affective disorder, post-traumatic stress disorder, or psychosis) will be excluded.

For peer review only - http://bmjopen.bmj.com/site/about/guidelines.xhtml

Interventions

The eligibility of psychological interventions will be based on a classification of evidence-based psychological interventions commonly used in systematic reviews of psychological interventions.[19] These include cognitive behavior therapy, behavioral activation therapy, problem-solving therapy, third-wave cognitive behavioral therapies, interpersonal psychotherapy, psychodynamic therapy, non-directive supportive therapy, and life review therapy. Such interventions include specific therapeutic techniques designed to improve mental health and psychological well-being (e.g., anxiety, depression, mental health-related quality of life, and psychological distress). Different intervention delivery modes (e.g., individual, group, a person with dementia-informal caregiver dyad, self-help), methods of support (e.g., e-mail, face-to-face, internet, telephone, video-conference), intervention setting, and professional background of the person supporting or delivering the intervention (e.g., nurses, psychologists, psychological practitioners) will be eligible for inclusion.

Comparators

Eligible study designs will allow for treatment effects to be isolated[48] with eligible comparator conditions based on standard definitions[49] and include: (1) no-treatment control; (2) wait-list control; (3) treatment as usual (TAU); (4) non-specific factors component control; (5) specific factors component control; and (6) active comparator.

Outcomes

Quantitative

Eligible studies will include one or more self-report, clinician, or proxy reports by an informal caregiver standardized primary outcome measurement of mental health or psychological well-being in the person with dementia or MCI, including: (1) anxiety; (2) depression; (3) mental-health related quality of life; (4); and psychological distress (defined as a measurement of non- symptoms associated with anxiety, depression, and stress).[50]

Qualitative

Qualitative studies eligible for inclusion will be: (1) studies that precede included RCTs conducted to inform intervention development; and (2) embedded studies within included RCTs that explore the acceptability (e.g., attitudes, barriers and facilitators to intervention use, perceived burden, perceived benefits, perceived effectiveness, perceived relevance, satisfaction, and understanding) of included psychological interventions from the perspective

For peer review only - http://bmjopen.bmj.com/site/about/guidelines.xhtml

of important stakeholders (e.g., people with dementia or MCI, informal caregivers, and healthcare professionals). If mixed-methods studies are identified that use quantitative measures of intervention acceptability (e.g., the Treatment Acceptability/Adherence Scale),[51] data will be extracted and triangulated with the qualitative findings.

Study designs

Quantitative

To examine intervention effectiveness, RCTs and cluster RCTs will be eligible for inclusion. Non-randomized controlled trials will be excluded. Given the likelihood that including studies of low quality,[52-55] or without a proper RCT design inflate the effect size,[26, 27] studies rated as high risk of bias regarding allocation and concealment randomization processes in accordance with the revised Cochrane risk-of-bias tool for randomized trials (RoB 2)[40] will be excluded.

Qualitative

To explore acceptability, studies using any type of qualitative research study design exploring the acceptability of interventions from the perspective of key stakeholders (e.g., people with dementia or MCI, informal caregivers, and healthcare professionals) will be eligible for inclusion.

Search methods

Electronic searches

A search strategy based on medical subject headings (MeSH) will be carried out in the following relevant international electronic databases: ASSIA, CENTRAL, CINAHL; EMBASE; PsychInfo; and MEDLINE. A draft of the MEDLINE search strategy can be found in Appendix I. Searches in the remaining electronic databases will be adapted to the syntax and subject headings. The search strategy was developed alongside Agnes Kotka (librarian at Uppsala University Library).

Other resources

Hand searches will supplement electronic searches (e.g., expert contact, back-forward citation, and reference checking) to identify additional RCTs potentially eligible for inclusion. Hand searches will also be used to search for qualitative research preceding and embedded

For peer review only - http://bmjopen.bmj.com/site/about/guidelines.xhtml

within included RCTs that explore the acceptability of included interventions. Grey literature will be searched using OpenGrey (http://www.opengrey.eu/).

No date restrictions will be imposed when searching electronic or other resources, however, due to resource limitations only papers published in English and Swedish will be included. Both published and unpublished studies will be eligible for inclusion, however, due to time constraints, we will not include full dissertations.

Screening and data extraction

Screening

Title and abstract screening, and full paper checking will be performed by two reviewers independently. Duplicate studies across searches will be identified and removed before title and abstract searching using EndNote X9 (Clarivate).[56] Titles and abstracts from retrieved citations will be screened independently by two reviewers using Rayyan.[57] Full papers of potentially eligible studies will be checked independently by two reviewers against the inclusion/exclusion criteria. The corresponding author will be emailed to resolve questions about eligibility when necessary, with a follow-up e-mail sent to corresponding authors who do not respond to the first e-mail within two weeks. Reasons for exclusion will be recorded and reported in a PRISMA flow chart. A detailed exclusion table will be presented for each specific inclusion/exclusion criterion in accordance with the PICOS statement. The two reviewers will discuss discrepancies, with a third reviewer consulted if needed.

Quantitative data extraction

Two reviewers will independently extract data from the included studies into Microsoft Excel. Reviewers will resolve disagreements by discussion, with a third reviewer adjudicating any disagreements. In case of any continued uncertainties, corresponding authors will be contacted by e-mail, with a follow-up e-mail for authors who do not respond to the first e-mail within two weeks. The following data will be extracted:

Study characteristics: aims and objectives, country of origin, funding, identification features (i.e., authors, citation details), inclusion/exclusion criteria, language, primary outcome measurements, publication type, and study design.

Participant characteristics: age, dementia subtype (e.g., Alzheimer's disease cerebrovascular/vascular disease, Frontotemporal dementia, Lewy body disease, or mixed), how dementia diagnosis was established, educational status, ethnicity, gender, provision of

For peer review only

313 support from an informal caregiver, severity of cognitive impairment and dementia, and time
314 since dementia diagnosis.
315 Intervention characteristics: extracted as per the Template for Intervention Description and
316 Replication (TIDieR) checklist[36] (Table 1).
317
318 **Table 1**. The Template for Intervention Description and Replication (TIDieR) checklist and
319 guide.[36]

Item Number	TIDieR Item	Brief Description
**1**	Brief name	Brief Name of Intervention: the name or phrase that describes the intervention
**2**	Why	Rationale, Theory, or Goal of the Intervention
**3**	What	Physical or Informational Materials: provided to participants or used in intervention delivery or in training intervention providers
**4**		Procedures, Activities, and Processes: including enabling or support activities
**5**	Who provided	Intervention Provider: including expertise, background, and specific training given
**6**	How	Mode of Delivery: e.g., face-to-face, internet, telephone, and whether provided individually or in a group
**7**	Where	Location: including any necessary infrastructure or relevant features
**8**	When and how much	Timing, Duration, and Intensity: number of times the intervention was delivered and over what period of time including the number of sessions, their schedule, and their duration, intensity or dose
**9**	Tailoring	Tailoring of Intervention: if planned to be personalised, titrated or adapted, then describe what, why, when, and how
**10**	Modification	Modification of Intervention: if modified during the course of the study, describe the changes (what, why, when, and how)
**11**	How well	Planned: if intervention adherence or fidelity was assessed, describe how and by whom, and if any strategies were used to maintain or improve fidelity
**12**		Actual: if intervention adherence or fidelity was assessed, describe the extent to which the intervention was delivered as planned

320
321

For peer review only - http://bmjopen.bmj.com/site/about/guidelines.xhtml

Recruitment characteristics: individual patient or dyad (i.e., the person with dementia and informal caregivers), recruitment setting, recruitment method, sampling method, and type of consent.

Participant flow: total number of people with dementia or MCI (and informal caregivers where applicable) invited, screened, eligible, and randomized, number randomized to each intervention arm. *For each intervention arm and time point:* number analyzed and lost to follow-up, with number attrition ≤ 6 months and percentage attrition ≤ 6 months calculated.

Results**:** Primary outcomes measurement (see Outcomes section for primary outcomes). *For each primary outcome and intervention arm:* time point, mean, SD (or standard error or confidence interval), and number of participants analyzed.

Intervention acceptability: intervention and study dropout rates, adherence to the intervention, quantitative measures of intervention acceptability and satisfaction (e.g., the Treatment Acceptability/Adherence Scale).[51]

Qualitative data extraction

Qualitative data from included studies will be imported into NVivo[58] to facilitate analysis. The following data will be extracted:

Study characteristics: standard study information including identification features, publication type, funding, language, aims and objectives, country of origin, funding, identification features (i.e., authors, citation details), language, and qualitative methodology.

Stakeholder characteristics: data describing the characteristics of important stakeholders (e.g., people with dementia or MCI, informal caregivers, and healthcare professionals).

Acceptability: qualitative data exploring the acceptability of interventions from the perspective of important stakeholders (e.g., attitudes, barriers and facilitators to intervention use, perceived burden, perceived benefits, perceived effectiveness, perceived relevance, satisfaction, and understanding).

Quality appraisal

Quantitative

Risk of bias

Risk of bias for each included study will be assessed independently by two reviewers using the Cochrane Collaboration's Risk of Bias tool 2.0 (RoB 2.0).[40] Published material (e.g., published paper, study protocol, and trial registration) and additional unpublished information from study authors will inform the assessment. We will contact study authors if there is

For peer review only - http://bmjopen.bmj.com/site/about/guidelines.xhtml

missing relevant information or discrepancies between preregistered information (study protocol, trial registration) and the published results paper. The following domains will be assessed: (1) randomization; (2) allocation to intervention; (3) adherence to intervention; (4) handling of missing outcome data; (5) measurement of outcome; and (6) selective outcome reporting. Two reviewers will discuss rating discrepancies, if a consensus cannot be reached a third reviewer will be consulted. Each domain can be rated as of low risk of bias, some concerns, or of high risk of bias in accordance with RoB 2.0 guidance and results will be reported descriptively with the Robvis tool[59] used to visualize risk of bias.

Confidence in evidence

The Grading of Recommendations Assessment, Development, and Evaluation (GRADE) tool[60] will be used to assess the confidence in evidence for the primary outcomes (mental health and psychological well-being (e.g., anxiety, depression, mental health-related quality of life, and psychological distress)) across studies.[61, 62] Confidence in evidence will be rated as very low, low, moderate, or high. RCTs receive an initial high rating but confidence in evidence is downgraded if rated as not having sufficient quality in any of the domains: within-study risk of bias, directness of evidence, heterogeneity, precision of effect estimates, and risk of publication bias.[60]

Qualitative

Two researchers will independently assess the methodological quality of included qualitative studies using the Critical Appraisal Skills Programme (CASP) checklist for qualitative studies.[63] Rating discrepancies will be discussed with a third reviewer consulted if a consensus is not reached. Results will be reported descriptively.

Data synthesis

Informed by the convergent segregated approach, quantitative and qualitative analyses and syntheses will first be conducted separately, followed by an integration of evidence derived from both syntheses.[47] Quantitative data will be extracted into Microsoft Excel, narratively synthesized, and if data allows analyzed using Comprehensive Meta-analysis (V.4). Qualitative data will be managed and analyzed using NVivo.[58]

Meta-analysis

For peer review only

If data permits, a meta-analysis will be conducted. Post-treatment between-group standardized mean effect sizes will be calculated (Hedges' g) to determine[64] adopting a random effects model for:

- Outcomes relating to mental health and psychological well-being (e.g., anxiety, depression, mental health-related quality of life, and psychological distress) separately for people with dementia or MCI.
- Outcomes relating to mental health and psychological well-being (e.g., anxiety, depression, mental health-related quality of life, and psychological distress) separately for informal caregivers.

In studies with multiple time points, a primary endpoint of ≤ 6 months will be adopted, reducing the potential bias associated with short-term post-treatment effects and the risk of elevated effect sizes.[44, 52, 65, 66] In accordance with the Cochrane Handbook,[46] studies where two intervention groups are eligible for inclusion, the control group sample size will be halved, and comparisons with each intervention group will be conducted separately. Where two control groups are compared to one intervention group, the intervention sample size will be halved and comparisons with each control group will be conducted separately.[67] A random-effects model will be used given anticipated heterogeneity due to variations in intervention and methodological components, and participant characteristics.[68] Cochran's Q statistic will be used to examine heterogeneity.[69] The proportion of total variability due to between-study heterogeneity will be measured using I^2 and the prediction interval will be adopted as an index of population dispersion.[70, 71]

Moderator analysis

If sufficient data from included RCTs allow, subgroup analyses will examine the potential moderating effects of:

- Intervention delivery mode (e.g., individual, person with dementia-informal caregiver dyad, group, self-help).
- Length of follow-up (e.g., post-treatment, 6-month follow-up, 12-month follow-up).
- Method of support (e.g., e-mail, face-to-face, internet, telephone, video-conference).
- Recruitment setting (e.g., clinical, community, mixed).
- Type of control condition (e.g., no-treatment control, wait-list control, TAU, non-specific factors component control, specific factors component control, active comparator).

For peer review only - http://bmjopen.bmj.com/site/about/guidelines.xhtml

- Type of dementia (e.g., Alzheimer's disease cerebrovascular/vascular disease, Frontotemporal dementia, Lewy body disease, or mixed).
- Type of psychological intervention (e.g., cognitive behavior therapy, behavioral activation therapy, problem-solving therapy, third-wave cognitive behavioral therapies, interpersonal psychotherapy, psychodynamic therapy, non-directive supportive therapy, life review therapy).

Sensitivity analyses

Sensitivity analysis regarding the overall effect size for each primary outcome (mental health and psychological well-being for people with dementia or MCI) will be conducted by temporarily removing: (1) each study individually; (2) studies with high attrition (≥ 30 % in at least one trial arm); (3) studies with a small sample size (≤ 20 in at least one trial arm);[54, 55] and (4) studies rated as high, moderate, and low risk of bias.

Funnel asymmetry

Funnel plot asymmetry will be examined for possible bias if there is a minimum of 10 studies in any meta-analysis. The estimated effect sizes, taking possible biases into account, will be calculated using the trim and fill procedure,[72] for each primary outcome (mental health and psychological well-being for people with dementia or MCI).

Intervention acceptability

A deductive framework synthesis will be adopted[73, 74] using the Theoretical Framework of Acceptability (TFA)[31] (Table 2). TFA constructs will be used as the deductive coding scheme[75] and inductive coding used for relevant data that does not fit with the TFA.[33] Full text included articles will be imported into NVivo 14 and data from the results sections and supplementary files will be coded in accordance with TFA constructs. Initially, a sample of 10% of included texts will be coded by two reviewers independently, with coding discussed in an analysis workshop with a third reviewer to facilitate conceptual consistency and develop a nuanced understanding of the data. The remaining data will be coded by one main reviewer, with analysis workshops held with the second reviewer to discuss and review the coding. Finally, a thematic analysis will be conducted by the main reviewer to identify themes that "go beyond" the primary data, synthesizing findings across studies in relation to review objectives. The thematic analysis will be subsequently discussed in an analysis workshop held with the second reviewer and will be peer-examined by other research team

For peer review only - http://bmjopen.bmj.com/site/about/guidelines.xhtml

456 members. Narrative descriptions of each theme will be provided, alongside supporting quotations. A review audit trail will be used to document all changes regarding codes, discussions, and decisions to increase trustworthiness.[76]

Table 2. The Theoretical Framework of Acceptability (TFA) [31].

TFA Construct	Definition
**Ethically**	The extent to which the intervention has good fit with an individual's value system
**Affective Attitude**	*Anticipated*: How an individual feel about the intervention prior to taking part<br>*Experienced*: How an individual feel about the intervention after taking part
**Burden**	*Anticipated*: The perceived amount of effort that is required to participate in the intervention<br>*Experienced*: The amount of effort that was required to participate in the intervention
**Opportunity Costs**	*Anticipated*: The extent to which benefits, profits, or values must be given up to engage in the intervention<br>*Experienced*: The benefits, profits or values that were given up to engage in the intervention
**Perceived Effectiveness**	*Anticipated*: The extent to which the intervention is perceived to be likely to achieve its purpose<br>*Experienced*: The extent to which the intervention is perceived to have achieved its intended purpose
**Self-efficacy**	The participant's confidence that they can perform the behaviour(s) required to participate in the intervention
**Intervention Coherence**	The extent to which the participant understands the intervention and how it works

Completeness and quality of intervention reporting

To determine the overall completeness and quality of intervention reporting, each item of the TIDieR checklist[36] (Table 1) will be scored as: 0 (not reported), 1 (partially reported), or 2 (fully reported), allowing a total summary score ranging from 0 (poor reporting) to 24 (full reporting).[77] Overall completeness of intervention reporting will be determined within each study, as well as across all studies.

Integration of quantitative and qualitative evidence

For peer review only - http://bmjopen.bmj.com/site/about/guidelines.xhtml

In accordance with the convergent segregated approach,[47] quantitative and qualitative findings will be integrated and synthesised once each separate synthesis has been conducted. Integration of quantitative and qualitative findings can result in a greater understanding and aid interpretation of results, compared to only undertaking two separate syntheses without formally linking the two sets of evidence.[47] Findings of each synthesis will be compared and contrasted accordingly[47] and presented either in a configured analysis or narrative analysis if configuration is not possible.[47]

Patient and Public Involvement

No patients or public were involved in the design or development of this protocol. However, we have already established a Public Advisory Board consisting of informal caregivers (n=4) with lived experience of caring for a person with dementia. The Public Advisory Board members are aged 44–71 years old, and are wives and daughters of people living with dementia with 5–9 years of experience caring for a person with dementia. The Public Advisory Board members will be presented with review findings to help aid interpretation and their perspectives will be incorporated into the results of the review.[78]

DISCUSSION

This is a study protocol for a mixed-methods systematic review that aims to: (1) examine the effectiveness of psychological interventions to improve mental health and psychological well-being in people with dementia or MCI; (2) examine the effectiveness of these psychological interventions to improve mental health and psychological well-being in informal caregivers; (3) examine potential clinical and methodological moderators associated with effectiveness; (4) explore factors associated with the acceptability of psychological interventions from the perspective of key stakeholders; and (5) examine the completeness and quality of intervention reporting.

This will review will extend the existing literature[13-15, 18] by including novel aspects such as narrowing the classification and definition of eligible psychological interventions, excluding studies with a high risk of bias, examining clinical and methodological moderators, and exploring intervention acceptability and the completeness and quality of intervention reporting. The planned review has additional strengths. The review adopts a mixed-method approach, allowing us to synthesize evidence from multiple sources to address review

For peer review only - http://bmjopen.bmj.com/site/about/guidelines.xhtml

questions. The review follows quality standards informed by the New Cochrane Handbook for Systematic Reviews of Interventions,[46] PRISMA 2020 guidelines,[43] and ENTREQ.[42] For example, screening, selection, data extraction, and quality appraisal will be assessed by two researchers independently. While there are several strengths, there are also limitations. Despite the CASP checklist being a recommended tool for assessing the quality of qualitative studies,[79] the checklist does not include a score rating system for scoring the quality of qualitative studies. This may lead to the inclusion of low-quality qualitative studies. However, to try to overcome this, two reviewers will rate the studies with a third reviewer consulted if a consensus cannot be reached, which may mitigate this problem. Due to limited resources, only studies available in English and Swedish language will be included, and language bias may be present.[80]

This review will be conducted within phase I (Development) of the Medical Research Council (MRC) framework for developing complex intervention guidance.[81] Results will be used to inform the further adaptation of a psychological intervention targeting depression among people with dementia[82] by identifying important considerations such as barriers and facilitators for intervention use, intervention acceptability, and potential intervention adaptations needed. Results will also provide important information regarding intervention components and intervention reporting quality and completeness of psychological interventions for people with dementia or MCI. Such findings will be useful for implementation considerations, and healthcare providers and policymakers delivering psychological interventions for people with dementia.

ETHICS AND DISSEMINATION

No ethical approval or informed consents will be required as no original data will be collected. Results will be published in an open access peer-reviewed journal, presented at academic conferences, and disseminated among lay and healthcare professional audiences.

LIST OF ABBREVIATIONS

BA: Behavioural activation

CASP: Critical Appraisal Skills Programme

CBT: Cognitive behavioral therapy

For peer review only - http://bmjopen.bmj.com/site/about/guidelines.xhtml

CRD: Centre for Reviews and Dissemination

ENTREQ: Enhancing Transparency in Reporting the Synthesis of Qualitative Research

MCI: Mild cognitive impairment

MeSH: Medical subject headings

MRC: Medical Research Council

PICOS: Participants, interventions, comparisons, outcomes, and study designs

PRISMA: Preferred Reporting Items for Systematic Reviews and Meta-Analyses

PRISMA-P: Preferred Reporting Items for Systematic Reviews and Meta-Analyses – Protocol

PRISMA-2020: Preferred Reporting Items for Systematic Reviews and Meta-Analyses 2020

PROSPERO: International prospective register of systematic reviews

TAU: Treatment as usual

TIDieR: Template for Intervention Description and Replication

RCT: Randomized controlled trial

DECLARATIONS

Ethics approval and consent to participate

Not applicable.

Consent for publication

Not applicable.

Availability of data and materials

Not applicable.

Competing interests

The authors declare that they have no competing interests.

Funding

For peer review only

This work was supported by the Swedish Research Council (Dnr: 2018-02691) and via the Swedish Research Council to U-CARE, a Strategic Research environment (Dnr: 2009–1093). Funders were not involved in the creation, development, or publication of this protocol. Funders will not be involved in the conduct, analysis, or reporting of the resulting study.

Authors' contributions

FS: Methodology, Resources, Writing – original draft, Visualization, Project Administration. **OB**: Methodology, Writing – review and editing, Project Administration. **PF**: Conceptualization, Methodology, Writing – review and editing, Supervision. **AB**: Writing – review and editing, Supervision. **LvE**: Writing—review and editing, Funding Acquisition. **ACÅ**: Writing – review and editing. **JW** (review guarantor): Conceptualization, Methodology, Writing – original draft, Supervision, Project Administration, Funding Acquisition.

Acknowledgments

We would like to thank Agnes Kotka, Librarian at Uppsala University Library for assisting with the development of the electronic search strategy.

Supplementary information

Appendix 1: PRISMA-P checklist

Appendix 2: MEDLINE electronic search strategy

For peer review only - http://bmjopen.bmj.com/site/about/guidelines.xhtml

REFERENCES

1 Bacsu JD, Johnson S, O'Connell ME, et al. Stigma reduction interventions of dementia: a scoping review. Can J Aging. 2022;41(2):203-13. doi:10.1017/S0714980821000192.

2 Livingston G, Huntley J, Sommerlad A, et al. Dementia prevention, intervention, and care: 2020 report of the Lancet Commission. Lancet. 2020;396(10248):413-46. doi:10.1016/S0140-6736(20)30367-6.

3 Taudorf L, Nørgaard A, Brodaty H, et al. Dementia increases mortality beyond effects of comorbid conditions: a national registry-based cohort study. Eur J Neurol. 2021;28(7):2174-84. doi:10.1111/ene.14875.

4 Smits LL, van Harten AC, Pijnenburg YA, et al. Trajectories of cognitive decline in different types of dementia. Psychol Med. 2015;45(5):1051-9. doi:10.1017/S0033291714002153.

5 Gill DP, Hubbard RA, Koepsell TD, et al. Differences in rate of functional decline across three dementia types. Alzheimers Dement. 2013;9(5Suppl):63-S71. doi:10.1016/j.jalz.2012.10.010.

6 Burks HB, des Bordes JKA, Chadha R, et al. Quality of life assessment in older adults with dementia: a systematic review. Dement Geriatr Cogn Disord. 2021;50(2):103-10. doi:10.1159/000515317.

7 GBD 2016 Dementia Collaborators. Global, regional, and national burden of Alzheimer's disease and other dementias, 1990-2016: a systematic analysis for the Global Burden of Disease Study 2016. Lancet Neurol. 2019;18(1):88-106. doi:10.1016/S1474-4422(18)30403-4.

8 Chen C, Hu Z, Jiang Z, et al. Prevalence of anxiety in patients with mild cognitive impairment: a systematic review and meta-analysis. J Affect Disord. 2018;236:211-21. doi:10.1016/j.jad.2018.04.110.

9 Ismail Z, Elbayoumi H, Fischer CE, et al. Prevalence of depression in patients with mild cognitive impairment: a systematic review and meta-analysis. JAMA Psychiatry. 2017;74(1):58-67. doi:10.1001/jamapsychiatry.2016.3162.

10 Leung DKY, Chan WC, Spector A, et al. Prevalence of depression, anxiety, and apathy symptoms across dementia stages: a systematic review and meta-analysis. Int J Geriatr Psychiatry. 2021;36(9):1330-44. doi:10.1002/gps.5556.

For peer review only

For peer review only - http://bmjopen.bmj.com/site/about/guidelines.xhtml

11 Mendez MF. The relationship between anxiety and alzheimer's disease. J Alzheimers Dis Rep. 2021;5(1):171-7. doi:10.3233/ADR-210294.

12 Kwak YT, Yang Y, Koo MS. Anxiety in dementia. Dement Neurocogn Disord. 2017;16(2):33-9. doi:10.12779/dnd.2017.16.2.33.

13 Na R, Yang JH, Yeom Y, et al. A systematic review and meta-analysis of nonpharmacological interventions for moderate to severe dementia. Psychiatry Investig. 2019;16(5):325-35. doi:10.30773/pi.2019.02.11.2.

14 Orgeta V, Leung P, Del-Pino-Casado R, et al. Psychological treatments for depression and anxiety in dementia and mild cognitive impairment. Cochrane Database Syst Rev. 2022;4(4):CD009125. doi:10.1002/14651858.CD009125.pub3.

15 Watt JA, Goodarzi Z, Veroniki AA, et al. Comparative efficacy of interventions for reducing symptoms of depression in people with dementia: systematic review and network meta-analysis. BMJ. 2021;372:n532. doi:10.1136/bmj.n532.

16 Black BS, Johnston D, Leoutsakos J, et al. Unmet needs in community-living persons with dementia are common, often non-medical and related to patient and caregiver characteristics. Int Psychogeriatr. 2019;31(11):1643-54. doi:10.1017/S1041610218002296.

17 Morrisby C, Joosten A, Ciccarelli M. Do services meet the needs of people with dementia and carers living in the community? A scoping review of the international literature. Int Psychogeriatr. 2018;30(1):5-14. doi:10.1017/S1041610217001491.

18 Noone D, Stott J, Aguirre E, et al. Meta-analysis of psychosocial interventions for people with dementia and anxiety or depression. Aging Ment Health. 2019;23(10):1282-91. doi:10.1080/13607863.2018.1495177.

19 Cuijpers P, Karyotaki E, de Wit L, et al. The effects of fifteen evidence-supported therapies for adult depression: a meta-analytic review. Psychother Res. 2020;30(3):279-93. doi:10.1080/10503307.2019.1649732.

20 Clare L, Nelis SM, Quinn C, et al. Improving the experience of dementia and enhancing active life--living well with dementia: study protocol for the IDEAL study. Health Qual Life Outcomes. 2014;12:164. doi:10.1186/s12955-014-0164-6.

21 Cipriani G, Lucetti C, Carlesi C, et al. Depression and dementia. A review. Eur Geriatr Med. 2015;6:479-86. doi:10.1016/j.eurger.2015.07.010.

22 Mohlman J, Bryant C, Lenze EJ, et al. Improving recognition of late life anxiety disorders in Diagnostic and Statistical Manual of Mental Disorders, Fifth Edition: observations and recommendations of the Advisory Committee to the Lifespan

For peer review only - http://bmjopen.bmj.com/site/about/guidelines.xhtml

Disorders Work Group. Int J Geriatr Psychiatry. 2012;27(6):549-56. doi:10.1002/gps.2752.

23 Collins RN, Kishita N. Prevalence of depression and burden among informal care-givers of people with dementia: a meta-analysis. Aging Soc. 2020;40(11):2355-92. https://doi.org/10.1017/S0144686X19000527.

24 Contreras ML, Mioshi E, Kishita N. Factors related to the quality of life in family carers of people with dementia: a meta-analysis. J Geriatr Psychiatry Neurol. 2021;34(5):482-500. doi:10.1177/0891988720924713.

25 Ferraris G, Fisher O, Lamura G, et al. Dyadic associations between perceived social support and psychological well-being in caregivers and older care recipients. J Fam Psychol. 2022;36(8):1397-1406. doi:10.1037/fam0001009.

26 Dechartres A, Trinquart L, Faber T, et al. Empirical evaluation of which trial characteristics are associated with treatment effect estimates. J Clin Epidemiol. 2016;77:24-37. doi:10.1016/j.jclinepi.2016.04.005.

27 Saltaji H, Armijo-Olivo S, Cummings GG, et al. Impact of Selection Bias on Treatment Effect Size Estimates in Randomized Trials of Oral Health Interventions: A Meta-epidemiological Study. J Dent Res. 2018;97(1):5-13. doi:10.1177/0022034517725049.

28 Collins KA, Westra HA, Dozois DJ, et al. Gaps in accessing treatment for anxiety and depression: challenges for the delivery of care. Clin Psychol Rev. 2004;24(5):583-616. doi:10.1016/j.cpr.2004.06.001.

29 Kenning C, Daker-White G, Blakemore A, et al. Barriers and facilitators in accessing dementia care by ethnic minority groups: a meta-synthesis of qualitative studies. BMC Psychiatry. 2017;17(1):316. doi:10.1186/s12888-017-1474-0.

30 Stephan A, Bieber A, Hopper L, et al. Barriers and facilitators to the access to and use of formal dementia care: findings of a focus group study with people with dementia, informal carers and health and social care professionals in eight European countries. BMC Geriatr. 2018;18(1):131. doi:10.1186/s12877-018-0816-1.

31 Sekhon M, Cartwright M, Francis JJ. Acceptability of healthcare interventions: an overview of reviews and development of a theoretical framework. BMC Health Serv Res. 2017;17(1):88. doi:10.1186/s12913-017-2031-8.

32 Kruse C, Fohn J, Wilson N, et al. Utilization barriers and medical outcomes commensurate with the use of telehealth among older adults: systematic review. JMIR Med Inform. 2020;8(8):e20359. doi:10.2196/20359.

For peer review only - http://bmjopen.bmj.com/site/about/guidelines.xhtml

33 Bartlett YK, Kenning C, Crosland J, et al. Understanding acceptability in the context of text messages to encourage medication adherence in people with type 2 diabetes. BMC Health Serv Res. 2021;21(1):608. doi:10.1186/s12913-021-06663-2.

34 Hommel KA, Hente E, Herzer M, et al. Telehealth behavioral treatment for medication nonadherence: a pilot and feasibility study. Eur J Gastroenterol Hepatol. 2013;25(4):469-73. doi:10.1097/MEG.0b013e32835c2a1b.

35 Seckler E, Regauer V, Rotter T, et al. Barriers to and facilitators of the implementation of multi-disciplinary care pathways in primary care: a systematic review. BMC Fam Pract. 2020;21(1):113. doi:10.1186/s12875-020-01179-w.

36 Hoffmann TC, Glasziou PP, Boutron I, et al. Better reporting of interventions: template for intervention description and replication (TIDieR) checklist and guide. BMJ. 2014;348:g1687. doi:10.1136/bmj.g1687.

37 Vassar M, Pollard J, Rorah D, et al. Assessment of the completeness of intervention reporting of randomized clinical trials for alcohol use disorders: effect of the TIDieR checklist and guide. Drug Alcohol Depend. 2020;208:107824. doi:10.1016/j.drugalcdep.2019.107824.

38 Stevens A, Shamseer L, Weinstein E, et al. Relation of completeness of reporting of health research to journals' endorsement of reporting guidelines: a systematic review. BMJ. 2014;348:g3804. doi:10.1136/bmj.g3804.

39 Anderson JM, Stafford A, Jellison S, et al. Intervention reporting of published trials is insufficient in orthopedic surgery journals: application of the template for intervention description and replication checklist. Arthrosc Sports Med Rehabil. 2021;3(3):e619-27. doi:10.1016/j.asmr.2020.09.019.

40 Sterne JAC, Savović J, Page MJ, et al. RoB 2: a revised tool for assessing risk of bias in randomised trials. BMJ. 2019;366:l4898. doi:10.1136/bmj.l4898.

41 Moher D, Shamseer L, Clarke M, et al. Preferred reporting items for systematic review and meta-analysis protocols (PRISMA-P) 2015 statement. Syst Rev. 2015;4(1):1. doi:10.1186/2046-4053-4-1.

42 Tong A, Flemming K, McInnes E, et al. Enhancing transparency in reporting the synthesis of qualitative research: ENTREQ. BMC Med Res Methodol. 2012;12:181. doi:10.1186/1471-2288-12-181.

43 Page MJ, McKenzie JE, Bossuyt PM, et al. The PRISMA 2020 statement: an updated guideline for reporting systematic reviews. BMJ. 2021;372:n71. doi:10.1136/bmj.n71.

For peer review only - http://bmjopen.bmj.com/site/about/guidelines.xhtml

44 Farrand P, Matthews J, Dickens C, et al. Psychological interventions to improve psychological well-being in people with dementia or mild cognitive impairment: systematic review and meta-analysis protocol. BMJ Open. 2016;6(1):e009713. doi:10.1136/bmjopen-2015-009713.

45 Centre for Reviews and Dissemination (CRD). Systematic reviews: CRD's guidance for undertaking reviews in health care. York: Centre for Reviews and Dissemination, University of York; 2009.

46 Higgins JP, Eldridge S, Li T. Including variants on randomized trials. In: Higgins JP, Thomas J, Chandler J, Cumpston M, Li T, Page MJ, editors, et al. Cochrane handbook for systematic reviews of interventions. New Jersey: John Wiley & Sons; 2019. doi:10.1002/9781119536604.ch23.

47 Lizarondo L, Stern C, Carrier J, et al. Chapter 8: Mixed methods systematic reviews. In: Aromataris E, Munn Z (Editors). JBI Manual for Evidence Synthesis. Adelaide: JBI 2020. doi:10.46658/JBIMES-20-09.

48 Evans SR. Fundamentals of clinical trial design. J Exp Stroke Transl Med. 2010;3:19-27. doi:10.6030/1939-067x-3.1.19.

49 Mohr DC, Spring B, Freedland KE, et al. The selection and design of control conditions for randomized controlled trials of psychological interventions. Psychother Psychosom. 2009;78:275-84. doi:10.1159/000228248.

50 Viertiö S, Kiviruusu O, Piirtola M, et al. Factors contributing to psychological distress in the working population, with a special reference to gender difference. BMC Public Health. 2021;21(1):611. doi:10.1186/s12889-021-10560-y.

51 Milosevic I, Levy HC, Alcolado GM, et al. The Treatment Acceptability/Adherence Scale: moving beyond the assessment of treatment effectiveness. Cogn Behav Ther. 2015;44(6):456-69. doi:10.1080/16506073.2015.1053407.

52 Farrand P, Woodford J. Impact of support on the effectiveness of written cognitive behavioural self-help: a systematic review and meta-analysis of randomised controlled trials. Clin Psychol Rev. 2013;33(1):182-95. doi:10.1016/j.cpr.2012.11.001.

53 Farrand P, Woodford J. Effectiveness of cognitive behavioural self-help for the treatment of depression and anxiety in people with long-term physical health conditions: a systematic review and meta-analysis of randomised controlled trials. Ann Behav Med. 2015;49(4):579-93. doi:10.1007/s12160-015-9689-0.

54 Mårtensson E, Blomberg O, Pettman D, et al. Psychological interventions for depression among informal caregivers of older adult populations: protocol of a

For peer review only

For peer review only - http://bmjopen.bmj.com/site/about/guidelines.xhtml

systematic review and meta-analysis of randomised controlled trials. BMJ Open. 2020;10(9):e036402. doi:10.1136/bmjopen-2019-036402.

55 Pettman D, O'Mahen H, Skoog Svanberg A, et al. Effectiveness and acceptability of cognitive-behavioural therapy based interventions for maternal peripartum depression: a systematic review, meta-analysis and thematic synthesis protocol. BMJ Open. 2019;9(12):e032659. doi:10.1136/bmjopen-2019-032659.

56 Bramer WM, Giustini D, de Jonge GB, et al. De-duplication of database search results for systematic reviews in EndNote [published correction appears in J Med Libr Assoc. 2017 Jan;105(1):111]. J Med Libr Assoc. 2016;104(3):240-3. doi:10.3163/1536-5050.104.3.014.

57 Ouzzani M, Hammady H, Fedorowicz Z, et al. Rayyan-a web and mobile app for systematic reviews. Syst Rev. 2016;5(1):210. doi:10.1186/s13643-016-0384-4.

58 QSR International Pty Ltd. NVivo qualitative data analysis software, Version 12. QSR International Pty Ltd, Victoria. 2021. http://www.qsrinternational.com/nvivo. Accessed 5 May 2023.

59 McGuinness LA, Higgins JPT. Risk-of-bias VISualization (robvis): An R package and shiny web app for visualizing risk-of-bias assessments. Res Synth Methods. 2021;12(1):55-61. doi:10.1002/jrsm.1411.

60 Schünemann HJ, Oxman AD, Brozek J, et al. Grading quality of evidence and strength of recommendations for diagnostic tests and strategies [published correction appears in BMJ. 2008 May 24;336(7654). doi:10.1136/bmj.a139. Schünemann, A Holger J [corrected to Schünemann, Holger J]]. BMJ. 2008;336(7653):1106-10. doi:10.1136/bmj.39500.677199.AE.

61 Guyatt GH, Oxman AD, Vist GE, et al. GRADE: an emerging consensus on rating quality of evidence and strength of recommendations. BMJ. 2008;336(7650):924-6. doi:10.1136/bmj.39489.470347.AD.

62 Ryan R, Hill S. How to grade the quality of the evidence. Cochrane Consum Commun Gr. 2016:1-25.

63 Critical Appraisal Skills Programme (CASP). CASP qualitative checklist. CASP. 2017. https://casp-uk.net/images/checklist/documents/CASP-Qualitative-Studies-Checklist/CASP-Qualitative-Checklist-2018_fillable_form.pdf. Accessed 5 May 2023.

64 Hedges V. Distribution theory for Glass's estimator of effect size and related estimators. J Educ Behav Stat. 1981;6(2):107-28. doi:10.2307/1164588.

For peer review only - http://bmjopen.bmj.com/site/about/guidelines.xhtml

65 Ekers D, Webster L, Van Straten A, et al. Behavioural activation for depression; an update of meta-analysis of effectiveness and sub group analysis. PLoS One. 2014;9(6):e100100. doi:10.1371/journal.pone.0100100.

66 Flückiger C, Del Re AC, Munder T, et al. Enduring effects of evidence-based psychotherapies in acute depression and anxiety disorders versus treatment as usual at follow-up - a longitudinal meta-analysis. Clin Psychol Rev. 2014;34(5):367-75. doi:10.1016/j.cpr.2014.05.001.

67 Simblett S, Birch J, Matcham F, et al. A systematic review and meta-analysis of e-mental health interventions to treat symptoms of posttraumatic stress. JMIR Ment Health. 2017;4(2):e14. doi:10.2196/mental.5558.

68 Schmidt FL, Oh IS, Hayes TL. Fixed- versus random-effects models in meta-analysis: model properties and an empirical comparison of differences in results. Br J Math Stat Psychol. 2009;62(Pt1):97-128. doi:10.1348/000711007X255327.

69 Higgins JP, Thompson SG. Quantifying heterogeneity in a meta-analysis. Stat Med. 2002;21(11):1539-58. doi:10.1002/sim.1186.

70 Borenstein M. In a meta-analysis, the I-squared statistic does not tell us how much the effect size varies. J Clin Epidemiol. 2022;152:281-4. doi:10.1016/j.jclinepi.2022.10.003.

71 Higgins JP, Thompson SG, Deeks JJ, et al. Measuring inconsistency in meta-analyses. BMJ. 2003;327(7414):557-60. doi:10.1136/bmj.327.7414.557.

72 Duval S, Tweedie R. Trim and fill: a simple funnel-plot-based method of testing and adjusting for publication bias in meta-analysis. Biometrics. 2000;56(2):455-63. doi:10.1111/j.0006-341x.2000.00455.x.

73 Bartlett YK, Kenning C, Crosland J, et al. Understanding acceptability in the context of text messages to encourage medication adherence in people with type 2 diabetes. BMC Health Serv Res. 2021;21(1):608. doi:10.1186/s12913-021-06663-2.

74 Paskins Z, Crawford-Manning F, Cottrell E, et al. Acceptability of bisphosphonates among patients, clinicians and managers: a systematic review and framework synthesis. BMJ Open. 2020;10(11):e040634. doi:10.1136/bmjopen-2020-040634.

75 Murphy AL, Gardner DM. Pharmacists' acceptability of a men's mental health promotion program using the Theoretical Framework of Acceptability. AIMS Public Health. 2019;6(2):195-208. doi:10.3934/publichealth.2019.2.195.

76 Creswell JW, Miller DL. Determining validity in qualitative inquiry. Theory into practice. 2000;39(3):124-30. doi:10.1207/s15430421tip3903_2.

For peer review only

For peer review only - http://bmjopen.bmj.com/site/about/guidelines.xhtml

77 Yamato TP, Maher CG, Saragiotto BT, et al. Rasch analysis suggested that items from the template for intervention description and replication (TIDieR) checklist can be summed to create a score. J Clin Epidemiol. 2018;101:28-34. doi:10.1016/j.jclinepi.2018.05.014.

78 Pollock A, Campbell P, Struthers C, et al. Stakeholder involvement in systematic reviews: a scoping review. Syst Rev. 2018;7(1):208. doi:10.1186/s13643-018-0852-0.

79 Ma LL, Wang YY, Yang ZH, et al. Methodological quality (risk of bias) assessment tools for primary and secondary medical studies: what are they and which is better? Mil Med Res. 2020;7(1):7. doi:10.1186/s40779-020-00238-8.

80 Morrison A, Polisena J, Husereau D, et al. The effect of English-language restriction on systematic review-based meta-analyses: a systematic review of empirical studies. Int J Technol Assess Health Care. 2012;28(2):138-44. doi:10.1017/S0266462312000086.

81 Skivington K, Matthews L, Simpson SA, et al. A new framework for developing and evaluating complex interventions: update of Medical Research Council guidance. BMJ. 2021;374:n2061. doi:10.1136/bmj.n2061.

82 Svedin F, Brantnell A, Farrand P, et al. Adapting a guided low-intensity behavioural activation intervention for people with dementia and depression in the Swedish healthcare context (INVOLVERA): a study protocol using codesign and participatory action research. BMJ Open. 2021;11(7):e048097. doi:10.1136/bmjopen-2020-048097.

For peer review only - http://bmjopen.bmj.com/site/about/guidelines.xhtml

PRISMA-P (Preferred Reporting Items for Systematic review and Meta-Analysis Protocols) 2015 checklist: recommended items to address in a systematic review protocol*

Section and topic	Item No	Checklist item	Reported on Page
**ADMINISTRATIVE INFORMATION**			
Title:			
Identification	1a	Identify the report as a protocol of a systematic review	Page 0
Update	1b	If the protocol is for an update of a previous systematic review, identify as such	N/A
Registration	2	If registered, provide the name of the registry (such as PROSPERO) and registration number	Page 1
Authors:			
Contact	3a	Provide name, institutional affiliation, e-mail address of all protocol authors; provide physical mailing address of corresponding author	Page 0
Contributions	3b	Describe contributions of protocol authors and identify the	Page 19
Amendments	4	If the protocol represents an amendment of a previously completed or published protocol, identify as such and list changes; otherwise, state plan for documenting important protocol amendments	N/A
Support:			
Sources	5a	Indicate sources of financial or other support for the review	Page 18-19
Sponsor	5b	Provide name for the review funder and/or sponsor	Page 18-19
Role of sponsor or funder	5c	Describe roles of funder(s), sponsor(s), and/or institution(s), if any, in developing the protocol	N/A
**INTRODUCTION**			
Rationale	6	Describe the rationale for the review in the context of what is already known	Page 3-5
Objectives	7	Provide an explicit statement of the question(s) the review will address with reference to participants, interventions, comparators, and outcomes (PICO)	Page 5-6

For peer review only

**METHODS**			
Eligibility criteria	8	Specify the study characteristics (such as PICO, study design, setting, time frame) and report characteristics (such as years considered, language, publication status) to be used as criteria for eligibility for the review	Page 6-8
Information sources	9	Describe all intended information sources (such as electronic databases, contact with study authors, trial registers or other grey literature sources) with planned dates of coverage	Page 8-9
Search strategy	10	Present draft of search strategy to be used for at least one electronic database, including planned limits, such that it could be repeated	Appendix 2
Study records:			
Data management	11a	Describe the mechanism(s) that will be used to manage records and data throughout the review	Page 9
Selection process	11b	State the process that will be used for selecting studies (such as two independent reviewers) through each phase of the review (that is, screening, eligibility and inclusion in meta-analysis)	Page 9-11
Data collection process	11c	Describe planned method of extracting data from reports (such as piloting forms, done independently, in duplicate), any processes for obtaining and confirming data from investigators	Page 9-11
Data items	12	List and define all variables for which data will be sought (such as PICO items, funding sources), any pre-planned data assumptions and simplifications	Page 9-11
Outcomes and prioritization	13	List and define all outcomes for which data will be sought, including prioritization of main and additional outcomes, with rationale	Page 7-8, 12-13
Risk of bias in individual studies	14	Describe anticipated methods for assessing risk of bias of individual studies, including whether this will be done at the outcome or study level, or both; state how this information will be used in data synthesis	Page 11-12
Data synthesis	15a	Describe criteria under which study data will be quantitatively synthesised	Page 12-15
	15b	If data are appropriate for quantitative synthesis, describe planned summary measures, methods of handling data and methods of combining data from studies, including any planned exploration of consistency (such as I^2, Kendall's τ)	Page 12-16

For peer review only

	15c	Describe any proposed additional analyses (such as sensitivity or subgroup analyses, meta-regression)	Page 14
	15d	If quantitative synthesis is not appropriate, describe the type of summary planned	Page 12-13
Meta-bias(es)	16	Specify any planned assessment of meta-bias(es) (such as publication bias across studies, selective reporting within studies)	Page 14
Confidence in cumulative evidence	17	Describe how the strength of the body of evidence will be assessed (such as GRADE)	Page 12

*** It is strongly recommended that this checklist be read in conjunction with the PRISMA-P Explanation and Elaboration (cite when available) for important clarification on the items. Amendments to a review protocol should be tracked and dated. The copyright for PRISMA-P (including checklist) is held by the PRISMA-P Group and is distributed under a Creative Commons Attribution Licence 4.0.**

From: Shamseer L, Moher D, Clarke M, Ghersi D, Liberati A, Petticrew M, Shekelle P, Stewart L, PRISMA-P Group. Preferred reporting items for systematic review and meta-analysis protocols (PRISMA-P) 2015: elaboration and explanation. BMJ. 2015 Jan 2;349(jan02 1):g7647.

For peer review only

Database: *Ovid MEDLINE*

Search nr	Term	Comment
Concept 1 – Population		
1	exp Dementia/<br>OR exp Cognition Disorders/<br>OR Cognitive Dysfunction/	[MeSH]
2	(dement* or Alzheimer* or lewy* or frontotemporal or FTD or FTLD or tvFTD or OBS or OBD or (lewy* adj2 bod*) or organic brain syndrome* or organic brain disease or organic brain disorder* or (ADRD or AAMI or AACD or MCI or A-MCI or N-MCI or M-MCI or aMCI or MCIa or CIND or MCD or MNC or MNCD or NCD) or ag* associated cogniti* decline or ag* associated memory impairment or mild cognitive impairment or neurocognitive disorder* or preclinical AD or pre-clinical AD or (pre adj clinical AD)).ti,ab.	[Title/Abstract]
3	1 OR 2	
Concept 2 – Intervention		
4	exp Counseling/<br>OR exp Behavior Therapy/<br>OR exp Psychotherapy/<br>OR exp Bibliotherapy/	[MeSH]
5	((non adj pharmacologic*) or non-pharmacologic* or nonpharmacologic* or cognitive restructuring or cognitive reframing or behavio* activation or activity scheduling or problem solving or (cCBT or iCBT or ehealth or e-health or teletherapy or telehealth) or (self adj help) or (self adj manag*) or (self adj administer*) or (psycho* adj therapy) or (cognitive adj2 therap*) or (behavio* adj2 therap*) or (CBT or psychotherapy or psychodynamic or counseling or counselling or psychoeducation* or psychosocial or psycho-social) or (group adj therap*) or (group adj treatment*) or (group adj intervention*) or (group adj support) or psycho education*).ti,ab.	[Title/Abstract]
6	4 OR 5	
Concept 3 – Outcome		
7	exp Depression/<br>OR exp Anxiety/<br>OR exp Anxiety Disorders/<br>OR exp Mood Disorders/<br>OR Affective Symptoms/<br>OR Psychological Distress/	[MeSH]
8	("quality of life" or (mental adj health) or (mental adj distress) or (mental adj illness) or (psycholo*	[Title/Abstract]

	adj distress) or (neuropsycholog* or neuropsychiatric) or (mood or emotion* or affective or wellbeing or well-being or distress) or (negative adj affect) or Depress* or Melanchol* or Dysphori* or (anxiety or anxious or stress or worr*) or (well adj being)).ti,ab.	
9	7 OR 8	
Concept 4 – Study design		
10	exp Randomized Controlled Trial/<br>OR exp Clinical Trial/<br>OR Meta-Analysis/<br>OR Random Allocation/	[MeSH]
11	(random* controlled trial* or (RCT or Trial or review or meta-analysis) or (random* adj allocat*)).ti,ab.	[Title/Abstract]
12	10 OR 11	
Final results	3 AND 6 AND 9 AND 12	

peer review only

BMJ Open

Effectiveness, acceptability, and completeness and quality of intervention reporting of psychological interventions for people with dementia or mild cognitive impairment: protocol for a mixed-methods systematic review

Journal:	*BMJ Open*
Manuscript ID	bmjopen-2023-077180.R1
Article Type:	Protocol
Date Submitted by the Author:	24-Oct-2023
Complete List of Authors:	Svedin, Frida; Uppsala University, Women's and Children's Health<br>Blomberg, Oscar; Uppsala University, Department of Women's and Children's Health<br>Farrand, Paul; University of Exeter, Clinical Education Development and Research (CEDAR)<br>Brantnell, Anders; Uppsala University, Civil and Industrial Engineering; Uppsala University Department of Women's and Children's Health<br>von Essen, Louise; Uppsala University, Department of Women's and Children's Health<br>Åberg, Anna ; Uppsala University, Department of Public Health and Caring Sciences; Dalarna University, School of Health and Welfare<br>Woodford, Joanne; Uppsala University, Department of Women's and Children's Health
**Primary Subject Heading**:	Mental health
Secondary Subject Heading:	Public health, Geriatric medicine
Keywords:	Dementia < NEUROLOGY, MENTAL HEALTH, PSYCHIATRY, Depression & mood disorders < PSYCHIATRY, Anxiety disorders < PSYCHIATRY, Old age psychiatry < PSYCHIATRY

SCHOLARONE™
Manuscripts

Effectiveness, acceptability, and completeness and quality of intervention reporting of psychological interventions for people with dementia or mild cognitive impairment: protocol for a mixed-methods systematic review

Frida SVEDIN[1] (frida.svedin@kbh.uu.se), Oscar BLOMBERG[1] (oscar.blomberg@kbh.uu.se), Paul FARRAND[3] (p.a.farrand@exeter.ac.uk), Anders BRANTNELL[1,2] (anders.brantnell@kbh.uu.se), Louise von ESSEN[1] (louise-von.essen@kbh.uu.se), Anna Cristina ÅBERG[4,5] (anna.cristina.aberg@pubcare.uu.se), Joanne WOODFORD[1] (joanne.woodford@kbh.uu.se)

Corresponding author

Dr Joanne Woodford, Healthcare Sciences and e-health, Department of Women's and Children's Health, Uppsala University, 751 85 Uppsala, Sweden. E-mail: joanne.woodford@kbh.uu.se. Telephone: +4618 471 35 64

Affiliations

[1]Healthcare Sciences and e-Health, Department of Women's and Children's Health, Uppsala University, 751 85 Uppsala, Sweden.

[2]Industrial Engineering and Management, Department of Civil and Industrial Engineering, Uppsala University, 751 21 Uppsala, Sweden.

[3]Clinical Education, Development and Research (CEDAR); Psychology, University of Exeter, Perry Road, Devon, EX4 4QG, United Kingdom.

[4]Clinical Geriatrics, Department of Public Health and Caring Sciences, Uppsala University, 751 85 Uppsala, Sweden.

[5]Medical Science, School of Health and Welfare, Dalarna University, 791 88 Falun, Sweden.

For peer review only

ABSTRACT

Introduction: Mental health difficulties such as anxiety and depression have negative impacts on psychological well-being, and are common in people with dementia and mild cognitive impairment. However, access to psychological treatments is limited. This mixed-method systematic review will: (1) examine the effectiveness of psychological interventions to improve mental health and psychological well-being in people with dementia or mild cognitive impairment; (2) examine the effectiveness of these psychological interventions to improve mental health and psychological well-being in informal caregivers; (3) examine potential clinical and methodological moderators associated with effectiveness; (4) explore factors associated with the acceptability of psychological interventions from the perspective of key stakeholders; and (5) examine the completeness and quality of intervention reporting.

Methods and analysis: Electronic databases (ASSIA, CENTRAL, CINAHL, EMBASE, PsychInfo, and MEDLINE) will be systematically searched and supplemented with expert contact, reference and citation checking, and grey literature searches. If possible, we will conduct a meta-analysis to examine the overall effectiveness of psychological interventions to improve mental health and psychological well-being in people with dementia or mild cognitive impairment and their informal caregivers; and examine potential clinical and methodological moderators associated with effectiveness. We will conduct a deductive framework synthesis, informed by the Theoretical Framework of Acceptability, to explore factors associated with the acceptability of psychological interventions from the perspective of key stakeholders. In accordance with JBI guidance, we will adopt a convergent segregated approach to data synthesis and integration of quantitative and qualitative findings. We will examine the completeness and quality of intervention reporting according to the Template for Intervention Description and Replication (TIDieR) checklist and guide.

Ethics and dissemination: No primary data will be collected and therefore ethical approval is not required. Results will be disseminated through a peer-reviewed publication, academic conferences, and plain language summaries.

PROSPERO registration number: CRD42023400514.

Key Words: Dementia; MCI; Psychological Well-being; Depression; Anxiety; Informal caregivers; Mixed-methods Review

For peer review only - http://bmjopen.bmj.com/site/about/guidelines.xhtml

Strengths and limitations of this study:

- This review will extend existing literature by including novel aspects such as narrowing the classification and definition of eligible psychological interventions, excluding studies with a high risk of bias, and exploring intervention acceptability and the completeness of intervention reporting.
- We will adopt a mixed-method approach informed by the convergent segregated approach, allowing us to synthesise evidence from multiple sources to address review questions.
- This review will follow quality standards informed by the New Cochrane Handbook for Systematic Reviews of Interventions, PRISMA 2020 guidelines, and Enhancing transparency in reporting the synthesis of qualitative research (ENTREQ).
- Despite the Critical Appraisal Skills Programme (CASP) checklist being a recommended tool for assessing the quality of qualitative studies, it does not include a quality score rating system. This may lead to including low-quality qualitative studies.
- Due to limited resources, only studies available in English and Swedish will be included.

For peer review only

INTRODUCTION

Dementia is a global public health priority,[1] with the number of people living with dementia estimated to rise from 50 million in 2016 to 152 million in 2050.[2] Dementia has several significant adverse impacts, including increased mortality,[3] cognitive [4] and functional decline,[5] and poor quality of life.[6] Significant demands are placed on informal caregivers (i.e., family and friends who provide unpaid care) and wider society.[7] People with dementia and mild cognitive impairment (MCI) commonly experience co-morbid mental health difficulties, such as anxiety and depression [8-11] which are associated with exacerbation of cognitive decline, poor quality of life, sleep difficulties, and reduction in activities of daily living.[12] For people with dementia, the overall pooled prevalence is estimated to be 39% for both anxiety and for depression,[10] and for people with MCI 21% for depression[8] and 32% for anxiety.[9] Despite research suggesting psychological interventions are effective,[13-15] access to evidence-based psychological support for people with dementia is limited.[16, 17]

Recent reviews suggest psychological interventions for anxiety and depression in dementia or MCI are probably effective.[13-15, 18] However, these reviews have included a broad range of psychosocial interventions (e.g., psychosocial interventions focusing on psychological and social factors, relaxation training therapies, music therapies, exercise, and massage) yielding large heterogeneity in intervention type. To facilitate subgroup analyses to explore the potential moderating effect of psychological intervention type, we intend to build upon previous reviews by using a narrower classification of psychological interventions.[19] Using a narrower classification and potentially reducing clinical heterogeneity may facilitate identifying clinical moderators rarely investigated such as intervention delivery model (e.g., individual, dyadic, group, or self-administered) and method of support (e.g., face-to-face, internet, telephone, or video-conference).

Further, previous reviews[13-15, 18] have not examined psychological interventions for psychological well-being in people with dementia or MCI.[20] Such interventions may be of particular importance given the challenges experienced in diagnosing people with dementia and depression due to overlapping symptomology between late-life depression and dementia.[21] Difficulties are also experienced in diagnosing anxiety, given people with dementia or MCI may present with anxiety symptoms inconsistent with standardized diagnostic criteria.[22] Additionally, informal caregivers are at an increased risk of experiencing difficulties with their mental health and psychological well-being, for example,

For peer review only - http://bmjopen.bmj.com/site/about/guidelines.xhtml

burden and depression,[23] and poor quality of life.[24] Given the dyadic interdependence between the care recipient and caregiver's psychological well-being,[25] it may be important to examine whether psychological interventions to improve mental health and psychological well-being in people with dementia or MCI also improve informal caregiver mental health and psychological well-being s. In a previous review examining the effect of psychological treatments for anxiety and depression for dementia and MCI a small number of studies were found that included informal caregiver outcomes.[14] However, how interventions aimed at improving psychological well-being or mental health-related quality of life in people with dementia and MCI may also impact informal caregiver mental health and psychological well-being warrants further investigation. Finally, existing reviews have included studies with non-randomised designs[13] or with a high risk of bias concerning randomization procedures.[15] Including these studies may result in an overestimation of treatment effect.[26, 27] Consequently, there is a need to conduct a systematic review excluding studies of low methodological quality.

In addition to the aforementioned gaps in the evidence base, people with dementia or MCI commonly experience barriers to accessing psychological support. Barriers include factors related to the individual (e.g., lack of time for treatment or geographic distance), the provider (e.g., lack of willingness to diagnose and treat mental health difficulties), and the healthcare system (e.g., limited availability of speciality mental health providers).[28] Barriers commonly experienced by people with dementia or MCI include social stigmatization at the individual level,[29] and lack of knowledge of mental health problems among healthcare providers[17, 30] at the provider level. An additional access barrier is intervention acceptability, i.e., the anticipated or experienced cognitive and emotional response to an intervention from the perspective of those receiving and delivering the intervention.[31] For example, lack of motivation and low patient interest represent common barriers to intervention access among older adults.[32] It is essential to consider acceptability when developing, evaluating, and implementing healthcare interventions[31, 33] given associations with intervention adherence[31] and positive clinical outcomes.[34] However, to date, little is known about the acceptability of psychological interventions for people with dementia or MCI, and other important stakeholders (e.g., informal caregivers and healthcare professionals). Considering factors associated with acceptability[35] may help improve treatment access and enhance the implementation potential of future developed interventions

For peer review only - http://bmjopen.bmj.com/site/about/guidelines.xhtml

by increasing intervention acceptance and relevance from the perspective of those receiving and delivering/implementing the intervention.

An additional barrier to treatment access may relate to the completeness and quality of the reporting of psychological interventions in the research literature. The completeness and quality of published intervention descriptions (i.e., descriptions of evaluated interventions that are detailed enough to facilitate replication) are commonly poor.[36, 37] Consequently, reliable intervention replication and/or implementation is difficult.[36] Ensuring the transparency and completeness of intervention reporting may facilitate the adoption of interventions by clinicians, informal caregivers, patients, and policy decision-makers,[38] and is essential for implementing interventions in practice.[39] Existing reviews suggest intervention components of psychological interventions for people with dementia or MCI are poorly reported.[14] However, to the best of our knowledge, there is no comprehensive review describing the quality and completeness of intervention reporting of psychological interventions for people with dementia or MCI.

In summary, this mixed-methods systematic review will address the aforementioned gaps in the literature by: (1) using a narrower and commonly used classification of psychological interventions,[19] to reduce clinical heterogeneity and facilitate comparison of findings and exploration of potential clinical moderators; (2) examining the effectiveness of psychological interventions for people with dementia or MCI on general psychological well-being and mental health-related quality of life; (3) examining the effectiveness of these psychological interventions to improve the mental health and psychological well-being in informal caregivers; (4) excluding studies with non-randomised and uncontrolled designs and RCTs with randomisation procedures providing a high risk of bias, in line with the Cochrane Collaboration's Risk of Bias tool 2.0 (RoB 2.0);[40] (5) exploring intervention acceptability from the perspective of important stakeholders (e.g., people with dementia or MCI, informal caregivers, and healthcare professionals); and (6) examining intervention reporting quality and completeness.

Objectives

This mixed-method systematic review will: (1) examine the effectiveness of psychological interventions to improve mental health and psychological well-being in people with dementia or mild cognitive impairment; (2) examine the effectiveness of these psychological

For peer review only - http://bmjopen.bmj.com/site/about/guidelines.xhtml

interventions to improve mental health and psychological well-being in informal caregivers; (3) examine potential clinical and methodological moderators associated with effectiveness; (4) explore factors associated with the acceptability of psychological interventions from the perspective of key stakeholders; and (5) examine the completeness and quality of intervention reporting.

METHODS AND ANALYSIS

The review will adopt a mixed methods approach with the protocol reported in accordance with the Preferred Reporting Items for Systematic Reviews and Meta-Analyses Protocols (PRISMA-P) statement[41] (Appendix 1) and Enhancing Transparency in Reporting the Synthesis of Qualitative Research (ENTERQ) statement (Appendix 1).[42] Study results will be reported following the Preferred Reporting Items for Systematic Reviews and Meta-Analyses 2020 (PRISMA 2020) guidelines.[43] This protocol has been registered with the International Prospective Register of Systematic Reviews (PROSPERO) registration number: CRD42023400514 and is partially informed by a previous review protocol.[44] Centre of Reviews and Dissemination (CRD) guidance for conducting systematic reviews,[45] the New Cochrane Handbook for Systematic Reviews of Interventions,[46] and JBI guidance for mixed methods systematic reviews will be followed.[47]

Inclusion and exclusion criteria

Participants

Adults diagnosed with dementia or MCI in accordance with a validated diagnostic criterion (e.g., the Diagnostic and Statistical Manual (DSM), International Classification of Diseases (ICD), other validated criteria), or recorded in medical records. Eligible dementia diagnoses include Alzheimer's disease and related dementias (e.g., cerebrovascular/vascular disease, Frontotemporal dementia, Lewy body disease, or mixed). Adults diagnosed with dementia primarily due to human immunodeficiency virus, Huntington's disease, Parkinson's disease, Prion disease, substance/medication use, traumatic brain injury, multiple sclerosis, or other medical conditions will be excluded. No restrictions will be placed on dementia severity, or time since diagnosis. Community and institutional-dwelling persons with dementia or MCI will be eligible for inclusion. Persons with comorbid severe mental health problems (e.g., bipolar affective disorder, post-traumatic stress disorder, or psychosis) will be excluded.

Interventions

For peer review only - http://bmjopen.bmj.com/site/about/guidelines.xhtml

Psychological intervention eligibility will be based on a classification of evidence-based psychological interventions commonly used in systematic reviews of psychological interventions.[19] These include cognitive behavioural therapy, behavioural activation therapy, problem-solving therapy, third-wave cognitive behavioural therapies, interpersonal psychotherapy, psychodynamic therapy, non-directive supportive therapy, and life review therapy. Such interventions include specific therapeutic techniques designed to improve mental health and psychological well-being (e.g., anxiety, depression, mental health-related quality of life, and psychological distress). Different intervention delivery modes (e.g., individual, group, a person with dementia-informal caregiver dyad, self-help), methods of support (e.g., e-mail, face-to-face, internet, telephone, video-conference), intervention setting, and professional background of the person supporting or delivering the intervention (e.g., nurses, psychologists, psychological practitioners) will be eligible for inclusion.

Comparators

Eligible study designs will allow for treatment effects to be isolated[48] with eligible comparator conditions based on standard definitions[49] and include: (1) no-treatment control; (2) wait-list control; (3) treatment as usual (TAU); (4) non-specific factors component control; (5) specific factors component control; and (6) active comparator.

Outcomes

Quantitative

Eligible studies will include one or more self-report, clinician, or proxy reports by an informal caregiver standardised primary outcome measurement of mental health or psychological well-being in the person with dementia or MCI, including: (1) anxiety; (2) depression; (3) mental-health related quality of life; (4); and psychological distress (defined as a measurement of non-symptoms associated with anxiety, depression, and stress).[50]

Qualitative

Qualitative studies eligible for inclusion will be: (1) studies that precede included RCTs conducted to inform intervention development; and (2) embedded studies within included RCTs that explore the acceptability (e.g., attitudes, barriers and facilitators to intervention use, perceived burden, perceived benefits, perceived effectiveness, perceived relevance, satisfaction, and understanding) of included psychological interventions from the perspective of important stakeholders (e.g., people with dementia or MCI, informal caregivers, and

For peer review only - http://bmjopen.bmj.com/site/about/guidelines.xhtml

healthcare professionals). If mixed-methods studies are identified using quantitative measures of intervention acceptability (e.g., the Treatment Acceptability/Adherence Scale),[51] data will be extracted and triangulated with the qualitative findings.

Study designs

Quantitative

To examine intervention effectiveness, RCTs and cluster RCTs will be eligible for inclusion. Non-randomised controlled trials will be excluded. Given the likelihood that including studies of low quality,[52-55] or without a proper RCT design inflates the effect size,[26, 27] studies rated as high risk of bias regarding allocation and concealment randomisation processes in accordance with the RoB 2 tool[40] will be excluded.

Qualitative

To explore acceptability, studies using any type of qualitative research study design exploring the acceptability of interventions from the perspective of key stakeholders (e.g., people with dementia or MCI, informal caregivers, and healthcare professionals) will be eligible for inclusion.

Search methods

Electronic searches

A search strategy based on medical subject headings (MeSH) will be carried out in the following relevant international electronic databases: ASSIA, CENTRAL, CINAHL, EMBASE, PsychInfo, and MEDLINE. A draft of the MEDLINE search strategy can be found in Appendix 2. Searches in the remaining electronic databases will be adapted to the syntax and subject headings. The search strategy was developed alongside a librarian at Uppsala University Library. Electronic databases will be searched from date of inception up to the date the search was conducted.

Other resources

Hand searches will supplement electronic searches (e.g., expert contact, back-forward citation, and reference checking) to identify additional RCTs potentially eligible for inclusion. Hand searches will also be used to search for qualitative research preceding and embedded within included RCTs that explore the acceptability of included interventions. Grey literature will be searched using OpenGrey (http://www.opengrey.eu/).

For peer review only - http://bmjopen.bmj.com/site/about/guidelines.xhtml

No date restrictions will be imposed when searching electronic or other resources, however, due to resource limitations only papers published in English and Swedish will be included. Both published and unpublished studies will be eligible for inclusion, however, due to time constraints, we will not include full dissertations.

Screening and data extraction

Screening

Title and abstract screening, and full paper checking will be performed by two reviewers independently. Duplicate studies across searches will be identified and removed before title and abstract searching using EndNote X9 (Clarivate).[56] Titles and abstracts from retrieved citations will be screened independently by two reviewers using Rayyan.[57] Full papers of potentially eligible studies will be checked independently by two reviewers against the inclusion/exclusion criteria. The two reviewers will discuss discrepancies, with a third reviewer consulted if needed. Corresponding authors will be e-mailed to resolve questions about eligibility when necessary, with a follow-up e-mail sent to corresponding authors who do not respond to the first e-mail within two weeks. Reasons for exclusion will be recorded and reported in a PRISMA flow chart. A detailed exclusion table will be presented for each specific inclusion/exclusion criterion in accordance with the PICOS statement.

Quantitative data extraction

Two reviewers will independently extract data from the included studies into Microsoft Excel. Reviewers will resolve disagreements by discussion, with a third reviewer adjudicating any disagreements. In case of any continued uncertainties, corresponding authors will be contacted by e-mail, with a follow-up e-mail for authors who do not respond to the first e-mail within two weeks. The following data will be extracted:

Study characteristics: aims and objectives, country of origin, funding, identification features (i.e., authors, citation details), inclusion/exclusion criteria, language, primary outcome measurements, publication type, and study design.

Participant characteristics: age, dementia subtype (e.g., Alzheimer's disease cerebrovascular/vascular disease, Frontotemporal dementia, Lewy body disease, or mixed), how dementia diagnosis was established, educational status, ethnicity, gender, provision of support from an informal caregiver, severity of cognitive impairment and dementia, and time since dementia diagnosis.

For peer review only - http://bmjopen.bmj.com/site/about/guidelines.xhtml

Intervention characteristics: extracted as per the Template for Intervention Description and Replication (TIDieR) checklist[36] (Table 1).

Table 1. The Template for Intervention Description and Replication (TIDieR) checklist and guide*.

Item Number	TIDieR Item	Brief Description
**1**	Brief name	Brief Name of Intervention: the name or phrase that describes the intervention
**2**	Why	Rationale, Theory, or Goal of the Intervention
**3**	What	Physical or Informational Materials: provided to participants or used in intervention delivery or in training intervention providers
**4**		Procedures, Activities, and Processes: including enabling or support activities
**5**	Who provided	Intervention Provider: including expertise, background, and specific training given
**6**	How	Mode of Delivery: e.g., face-to-face, internet, telephone, and whether provided individually or in a group
**7**	Where	Location: including any necessary infrastructure or relevant features
**8**	When and how much	Timing, Duration, and Intensity: number of times the intervention was delivered and over what period of time including the number of sessions, their schedule, and their duration, intensity or dose
**9**	Tailoring	Tailoring of Intervention: if planned to be personalised, titrated or adapted, then describe what, why, when, and how
**10**	Modification	Modification of Intervention: if modified during the course of the study, describe the changes (what, why, when, and how)
**11**	How well	Planned: if intervention adherence or fidelity was assessed, describe how and by whom, and if any strategies were used to maintain or improve fidelity
**12**		Actual: if intervention adherence or fidelity was assessed, describe the extent to which the intervention was delivered as planned

Adapted from Hoffmann et al. 2014: Better reporting of interventions: template for intervention description and replication (TIDieR) checklist and guide.[36]

For peer review only

For peer review only - http://bmjopen.bmj.com/site/about/guidelines.xhtml

Recruitment characteristics: recruitment of individual persons with dementia or MCI or dyads (i.e., the person with dementia or MCI and informal caregiver), recruitment setting, recruitment method, sampling method, and type of consent.

Participant flow: total number of people with dementia or MCI (and informal caregivers where applicable) invited, screened, eligible, and randomised, number randomised to each intervention arm. *For each intervention arm and time point:* number analysed and lost to follow-up, with number attrition ≤ 6 months and percentage attrition ≤ 6 months calculated.

Results: Primary outcomes measurement (see Outcomes section for primary outcomes). *For each primary outcome and intervention arm:* time point, mean, SD (or standard error or confidence interval), and number of participants analysed.

Intervention acceptability: intervention and study dropout rates, adherence to the intervention, quantitative measures of intervention acceptability and satisfaction (e.g., the Treatment Acceptability/Adherence Scale).[51]

Qualitative data extraction

Qualitative data from included studies will be imported into NVivo[58] to facilitate analysis. The following data will be extracted:

Study characteristics: standard study information including identification features, publication type, funding, language, aims and objectives, country of origin, funding, identification features (i.e., authors, citation details), language, and qualitative methodology.

Stakeholder characteristics: data describing the characteristics of important stakeholders (e.g., people with dementia or MCI, informal caregivers, and healthcare professionals).

Acceptability: qualitative data exploring the acceptability of interventions from the perspective of important stakeholders (e.g., attitudes, barriers and facilitators to intervention use, perceived burden, perceived benefits, perceived effectiveness, perceived relevance, satisfaction, and understanding).

Quality appraisal

Quantitative

Risk of bias

Risk of bias for each included study will be assessed independently by two reviewers using the Cochrane Collaboration's Risk of Bias tool 2.0 (RoB 2.0).[40] Published material (e.g., published paper, study protocol, and trial registration) and additional unpublished information from study authors will inform the assessment. We will contact study authors if there is

For peer review only - http://bmjopen.bmj.com/site/about/guidelines.xhtml

missing relevant information or discrepancies between preregistered information (study protocol, trial registration) and the published results paper. The following domains will be assessed: (1) randomisation; (2) allocation to intervention; (3) adherence to intervention; (4) handling of missing outcome data; (5) measurement of outcome; and (6) selective outcome reporting. Two reviewers will discuss rating discrepancies, if a consensus cannot be reached a third reviewer will be consulted. Each domain can be rated as of low risk of bias, some concerns, or of high risk of bias in accordance with RoB 2.0 guidance and results will be reported descriptively with the Robvis tool[59] used to visualise risk of bias.

Confidence in evidence

The Grading of Recommendations Assessment, Development, and Evaluation (GRADE) tool[60] will be used to assess the confidence in evidence for the primary outcomes (mental health and psychological well-being (e.g., anxiety, depression, mental health-related quality of life, and psychological distress)) across studies.[61, 62] Confidence in evidence will be rated as very low, low, moderate, or high. RCTs receive an initial high rating but confidence in evidence is downgraded if rated as not having sufficient quality in any of the five domains: (1) within-study risk of bias; (2) directness of evidence; (3) heterogeneity; (4) precision of effect estimates, and (5) risk of publication bias.[60]

Qualitative

Two researchers will independently assess the methodological quality of included qualitative studies using the Critical Appraisal Skills Programme (CASP) checklist for qualitative studies.[63] Rating discrepancies will be discussed with a third reviewer consulted if consensus is not reached. Results will be reported descriptively.

Data synthesis

Informed by the convergent segregated approach, quantitative and qualitative analyses and syntheses will first be conducted separately, followed by an integration of evidence derived from both syntheses.[47] Quantitative data will be extracted into Microsoft Excel, narratively synthesized, and if data allows analysed using Comprehensive Meta-analysis (V.4). Qualitative data will be managed and analysed using NVivo.[58]

Meta-analysis

For peer review only - http://bmjopen.bmj.com/site/about/guidelines.xhtml

If data permits, a meta-analysis will be conducted. Post-treatment between-group standardised mean effect sizes will be calculated (Hedges' g) [64] adopting a random effects model for:

- Outcomes relating to mental health and psychological well-being (e.g., anxiety, depression, mental health-related quality of life, and psychological distress) separately for people with dementia or MCI.
- Outcomes relating to mental health and psychological well-being (e.g., anxiety, depression, mental health-related quality of life, and psychological distress) separately for informal caregivers.

In studies with multiple time points, a primary endpoint of ≤ 6 months will be adopted, reducing the potential bias associated with short-term post-treatment effects and the risk of elevated effect sizes.[44, 52, 65, 66] In accordance with the Cochrane Handbook,[46] studies where two intervention groups are eligible for inclusion, the control group sample size will be halved, and comparisons with each intervention group will be conducted separately. Where two control groups are compared to one intervention group, the intervention sample size will be halved and comparisons with each control group will be conducted separately.[67] A random-effects model will be used given anticipated heterogeneity due to variations in intervention and methodological components, and participant characteristics.[68] Cochran's Q statistic will be used to examine heterogeneity.[69] The proportion of total variability due to between-study heterogeneity will be measured using I^2 and the prediction interval will be adopted as an index of population dispersion.[70, 71]

Moderator analysis

If sufficient data from included RCTs allow, subgroup analyses will examine the potential moderating effects of:

- Intervention delivery mode (e.g., individual, person with dementia-informal caregiver dyad, group, self-help).
- Length of follow-up (e.g., post-treatment, 6-month follow-up, 12-month follow-up).
- Method of support (e.g., e-mail, face-to-face, internet, telephone, video-conference).
- Recruitment setting (e.g., clinical, community, mixed).
- Type of control condition (e.g., no-treatment control, wait-list control, TAU, non-specific factors component control, specific factors component control, active comparator).

For peer review only - http://bmjopen.bmj.com/site/about/guidelines.xhtml

- Type of dementia (e.g., Alzheimer's disease, cerebrovascular/vascular disease, Frontotemporal dementia, Lewy body disease, or mixed).
- Type of psychological intervention (e.g., cognitive behavior therapy, behavioral activation therapy, problem-solving therapy, third-wave cognitive behavioral therapies, interpersonal psychotherapy, psychodynamic therapy, non-directive supportive therapy, life review therapy).

Sensitivity analyses

Sensitivity analysis regarding the overall effect size for each primary outcome (mental health and psychological well-being for people with dementia or MCI) will be conducted by temporarily removing: (1) each study individually; (2) studies with high attrition (≥ 30 % in at least one trial arm); (3) studies with a small sample size (≤ 20 in at least one trial arm);[54, 55] and (4) studies rated as high, moderate, and low risk of bias.

Funnel asymmetry

Funnel plot asymmetry will be examined for possible bias if there is a minimum of 10 studies in any meta-analysis. Estimated effect sizes, taking possible biases into account, will be calculated using the trim and fill procedure,[72] for each primary outcome (mental health and psychological well-being for people with dementia or MCI).

Intervention acceptability

A deductive framework synthesis will be adopted[73, 74] using the Theoretical Framework of Acceptability (TFA)[31] (Table 2). TFA constructs will be used as the deductive coding scheme[75] and inductive coding used for relevant data that does not fit with the TFA.[33] Full text included articles will be imported into NVivo 14 and data from the results sections and relevant supplementary files will be coded in accordance with TFA constructs. Initially, a sample of 10% of included texts will be coded by two reviewers independently, with coding discussed in an analysis workshop with a third reviewer to facilitate conceptual consistency and develop a nuanced understanding of the data. Remaining data will be coded by one main reviewer, with analysis workshops held with the second reviewer to discuss and review the coding. Finally, a thematic analysis will be conducted by the main reviewer to identify themes that "go beyond" the primary data, sythesising findings across studies in relation to review objectives. The thematic analysis will be subsequently discussed in an analysis workshop held with the second reviewer and will be peer-examined by other research team members.

For peer review only - http://bmjopen.bmj.com/site/about/guidelines.xhtml

Narrative descriptions of each theme will be provided, alongside supporting quotations. A review audit trail will be used to document all changes regarding codes, discussions, and decisions to increase trustworthiness.[76]

Table 2. The Theoretical Framework of Acceptability (TFA)*.

TFA Construct	Definition
**Ethically**	The extent to which the intervention has a good fit with an individual's value system
**Affective Attitude**	*Anticipated*: How an individual feels about the intervention prior to taking part<br>*Experienced*: How an individual feels about the intervention after taking part
**Burden**	*Anticipated*: The perceived amount of effort that is required to participate in the intervention<br>*Experienced*: The amount of effort that was required to participate in the intervention
**Opportunity Costs**	*Anticipated*: The extent to which benefits, profits, or values must be given up to engage in the intervention<br>*Experienced*: The benefits, profits or values that were given up to engage in the intervention
**Perceived Effectiveness**	*Anticipated*: The extent to which the intervention is perceived to be likely to achieve its purpose<br>*Experienced*: The extent to which the intervention is perceived to have achieved its intended purpose
**Self-efficacy**	The participant's confidence that they can perform the behaviour(s) required to participate in the intervention
**Intervention Coherence**	The extent to which the participant understands the intervention and how it works

Adapted from Sekhon et al. 2017: Acceptability of healthcare interventions: an overview of reviews and development of a theoretical framework.[31]

Completeness and quality of intervention reporting

To determine the overall completeness and quality of intervention reporting, each item of the TIDieR checklist[36] (Table 1) will be scored as: 0 (not reported), 1 (partially reported), or 2 (fully reported), allowing a total summary score ranging from 0 (poor reporting) to 24 (full reporting).[77] Overall completeness of intervention reporting will be determined within each study, as well as across all studies.

For peer review only - http://bmjopen.bmj.com/site/about/guidelines.xhtml

Integration of quantitative and qualitative evidence

In accordance with the convergent segregated approach,[47] quantitative and qualitative findings will be integrated and synthesised once each separate synthesis has been conducted. Integration of quantitative and qualitative findings can result in a greater understanding and aid interpretation of results, compared to only undertaking two separate syntheses without formally linking the two sets of evidence.[47] Findings of each synthesis will be compared and contrasted accordingly[47] and presented either in a configured analysis or narrative analysis if configuration is not possible.[47]

Patient and Public Involvement

No patients or public were involved in the design or development of this protocol. However, we have already established a Public Advisory Board consisting of informal caregivers (n=4) with lived experience of caring for a person with dementia. The Public Advisory Board members are aged 44–71 years old, and are wives and daughters of people living with dementia with 5–9 years of experience caring for a person with dementia. The Public Advisory Board members will be presented with review findings in an online workshop to help aid interpretation and facilitate the research team to place review findings in context and their perspectives will be incorporated into the results of the review.[78] After presenting review findings, Public Advisory Board members will be asked to discuss (1) to what extent the framework synthesis of intervention acceptability reflects their own experiences; (2) whether the research team's interpretation of review findings is appropriate and/or whether findings might be interpreted in different ways; and (3) the implications of review findings for people with dementia and MCI and their informal caregivers. The perspectives of Public Advisory Board members perspectives will be incorporated into the results (i.e., reflections on the thematic synthesis) and discussion (i.e., interpretation of findings and implications) sections of the results manuscript as relevant.

DISCUSSION

This is a study protocol for a mixed-methods systematic review that aims to: (1) examine the effectiveness of psychological interventions to improve mental health and psychological well-being in people with dementia or MCI; (2) examine the effectiveness of these psychological interventions to improve mental health and psychological well-being in informal caregivers;

For peer review only - http://bmjopen.bmj.com/site/about/guidelines.xhtml

500 (3) examine potential clinical and methodological moderators associated with effectiveness;
501 (4) explore factors associated with the acceptability of psychological interventions from the
502 perspective of key stakeholders; and (5) examine the completeness and quality of intervention
503 reporting.

504

505 This review will extend the existing literature[13-15, 18] by including novel aspects such as
506 narrowing the classification and definition of eligible psychological interventions, excluding
507 studies with a high risk of bias, examining clinical and methodological moderators, and
508 exploring intervention acceptability and the completeness and quality of intervention
509 reporting. The planned review has additional strengths. We adopt a mixed-method approach,
510 allowing the synthesis of evidence from multiple sources to address review questions. We
511 follow quality standards informed by the New Cochrane Handbook for Systematic Reviews of
512 Interventions,[46] PRISMA 2020 guidelines,[43] and ENTREQ.[42] For example, screening,
513 selection, data extraction, and quality appraisal will be assessed by two researchers
514 independently. While there are several strengths, there are also limitations. Despite the CASP
515 checklist being a recommended tool for assessing the quality of qualitative studies,[79] the
516 checklist does not include a score rating system for scoring the quality of qualitative studies.
517 This may lead to the inclusion of low-quality qualitative studies. However, two reviewers will
518 rate the studies with a third reviewer consulted if a consensus cannot be reached, potentially
519 mitigating this limitation. Due to limited resources, only studies available in English and
520 Swedish will be included, and language bias may be present.[80]

521

522 This review will be conducted within phase I (Development) of the Medical Research Council
523 (MRC) framework for developing complex interventions.[81] Results will be used to inform
524 the further adaptation of a psychological intervention targeting depression among people with
525 dementia[82] by identifying important considerations such as barriers and facilitators for
526 intervention use, intervention acceptability, and potential intervention adaptations. Results
527 will also provide important information regarding intervention components and intervention
528 reporting quality and completeness of psychological interventions for people with dementia or
529 MCI. Such findings will be useful for implementation considerations, including healthcare
530 providers delivering psychological interventions for people with dementia and policymakers.

531

532 **ETHICS AND DISSEMINATION**

For peer review only - http://bmjopen.bmj.com/site/about/guidelines.xhtml

No ethical approval or informed consent will be required as no original data will be collected. Results will be published in an open-access peer-reviewed journal, presented at academic conferences, and disseminated among lay and healthcare professional audiences.

LIST OF ABBREVIATIONS

BA: Behavioural activation

CASP: Critical Appraisal Skills Programme

CBT: Cognitive behavioural therapy

CRD: Centre for Reviews and Dissemination

ENTREQ: Enhancing Transparency in Reporting the Synthesis of Qualitative Research

MCI: Mild cognitive impairment

MeSH: Medical subject headings

MRC: Medical Research Council

PICOS: Participants, interventions, comparisons, outcomes, and study designs

PRISMA: Preferred Reporting Items for Systematic Reviews and Meta-Analyses

PRISMA-P: Preferred Reporting Items for Systematic Reviews and Meta-Analyses – Protocol

PRISMA-2020: Preferred Reporting Items for Systematic Reviews and Meta-Analyses 2020

PROSPERO: International prospective register of systematic reviews

TAU: Treatment as usual

TIDieR: Template for Intervention Description and Replication

RCT: Randomised controlled trial

DECLARATIONS

Ethics approval and consent to participate

Not applicable.

For peer review only

Consent for publication

Not applicable.

Availability of data and materials

Not applicable.

Competing interests

The authors declare that they have no competing interests.

Funding

This work was supported by the Swedish Research Council (Dnr: 2018-02691) and via the Swedish Research Council to U-CARE, a Strategic Research environment (Dnr: 2009–1093). Funders were not involved in the creation, development, or publication of this protocol. Funders will not be involved in the conduct, analysis, or reporting of the resulting study.

Authors' contributions

FS: Methodology, Resources, Writing – original draft, Visualisation, Project Administration. **OB**: Methodology, Writing – review and editing, Project Administration. **PF**: Conceptualisation, Methodology, Writing – review and editing, Supervision. **AB**: Writing – review and editing, Supervision. **LvE**: Writing—review and editing, Funding Acquisition. **ACÅ**: Writing – review and editing. **JW** (review guarantor): Conceptualisation, Methodology, Writing – original draft, Supervision, Project Administration, Funding Acquisition.

Acknowledgements

We would like to thank Agnes Kotka, Librarian at Uppsala University Library for assisting with the development of the electronic search strategy.

Supplementary information

Appendix 1: PRISMA-P checklist and ENTERQ checklist

Appendix 2: MEDLINE electronic search strategy

For peer review only

For peer review only - http://bmjopen.bmj.com/site/about/guidelines.xhtml

REFERENCES

1 Bacsu JD, Johnson S, O'Connell ME, et al. Stigma reduction interventions of dementia: a scoping review. Can J Aging. 2022;41(2):203-13. doi:10.1017/S0714980821000192.

2 Livingston G, Huntley J, Sommerlad A, et al. Dementia prevention, intervention, and care: 2020 report of the Lancet Commission. Lancet. 2020;396(10248):413-46. doi:10.1016/S0140-6736(20)30367-6.

3 Taudorf L, Nørgaard A, Brodaty H, et al. Dementia increases mortality beyond effects of comorbid conditions: a national registry-based cohort study. Eur J Neurol. 2021;28(7):2174-84. doi:10.1111/ene.14875.

4 Smits LL, van Harten AC, Pijnenburg YA, et al. Trajectories of cognitive decline in different types of dementia. Psychol Med. 2015;45(5):1051-9. doi:10.1017/S0033291714002153.

5 Gill DP, Hubbard RA, Koepsell TD, et al. Differences in rate of functional decline across three dementia types. Alzheimers Dement. 2013;9(5Suppl):63-S71. doi:10.1016/j.jalz.2012.10.010.

6 Burks HB, des Bordes JKA, Chadha R, et al. Quality of life assessment in older adults with dementia: a systematic review. Dement Geriatr Cogn Disord. 2021;50(2):103-10. doi:10.1159/000515317.

7 GBD 2016 Dementia Collaborators. Global, regional, and national burden of Alzheimer's disease and other dementias, 1990-2016: a systematic analysis for the Global Burden of Disease Study 2016. Lancet Neurol. 2019;18(1):88-106. doi:10.1016/S1474-4422(18)30403-4.

8 Chen C, Hu Z, Jiang Z, et al. Prevalence of anxiety in patients with mild cognitive impairment: a systematic review and meta-analysis. J Affect Disord. 2018;236:211-21. doi:10.1016/j.jad.2018.04.110.

9 Ismail Z, Elbayoumi H, Fischer CE, et al. Prevalence of depression in patients with mild cognitive impairment: a systematic review and meta-analysis. JAMA Psychiatry. 2017;74(1):58-67. doi:10.1001/jamapsychiatry.2016.3162.

10 Leung DKY, Chan WC, Spector A, et al. Prevalence of depression, anxiety, and apathy symptoms across dementia stages: a systematic review and meta-analysis. Int J Geriatr Psychiatry. 2021;36(9):1330-44. doi:10.1002/gps.5556.

For peer review only - http://bmjopen.bmj.com/site/about/guidelines.xhtml

11 Mendez MF. The relationship between anxiety and alzheimer's disease. J Alzheimers Dis Rep. 2021;5(1):171-7. doi:10.3233/ADR-210294.

12 Kwak YT, Yang Y, Koo MS. Anxiety in dementia. Dement Neurocogn Disord. 2017;16(2):33-9. doi:10.12779/dnd.2017.16.2.33.

13 Na R, Yang JH, Yeom Y, et al. A systematic review and meta-analysis of nonpharmacological interventions for moderate to severe dementia. Psychiatry Investig. 2019;16(5):325-35. doi:10.30773/pi.2019.02.11.2.

14 Orgeta V, Leung P, Del-Pino-Casado R, et al. Psychological treatments for depression and anxiety in dementia and mild cognitive impairment. Cochrane Database Syst Rev. 2022;4(4):CD009125. doi:10.1002/14651858.CD009125.pub3.

15 Watt JA, Goodarzi Z, Veroniki AA, et al. Comparative efficacy of interventions for reducing symptoms of depression in people with dementia: systematic review and network meta-analysis. BMJ. 2021;372:n532. doi:10.1136/bmj.n532.

16 Black BS, Johnston D, Leoutsakos J, et al. Unmet needs in community-living persons with dementia are common, often non-medical and related to patient and caregiver characteristics. Int Psychogeriatr. 2019;31(11):1643-54. doi:10.1017/S1041610218002296.

17 Morrisby C, Joosten A, Ciccarelli M. Do services meet the needs of people with dementia and carers living in the community? A scoping review of the international literature. Int Psychogeriatr. 2018;30(1):5-14. doi:10.1017/S1041610217001491.

18 Noone D, Stott J, Aguirre E, et al. Meta-analysis of psychosocial interventions for people with dementia and anxiety or depression. Aging Ment Health. 2019;23(10):1282-91. doi:10.1080/13607863.2018.1495177.

19 Cuijpers P, Karyotaki E, de Wit L, et al. The effects of fifteen evidence-supported therapies for adult depression: a meta-analytic review. Psychother Res. 2020;30(3):279-93. doi:10.1080/10503307.2019.1649732.

20 Clare L, Nelis SM, Quinn C, et al. Improving the experience of dementia and enhancing active life--living well with dementia: study protocol for the IDEAL study. Health Qual Life Outcomes. 2014;12:164. doi:10.1186/s12955-014-0164-6.

21 Cipriani G, Lucetti C, Carlesi C, et al. Depression and dementia. A review. Eur Geriatr Med. 2015;6:479-86. doi:10.1016/j.eurger.2015.07.010.

22 Mohlman J, Bryant C, Lenze EJ, et al. Improving recognition of late life anxiety disorders in Diagnostic and Statistical Manual of Mental Disorders, Fifth Edition: observations and recommendations of the Advisory Committee to the Lifespan

For peer review only - http://bmjopen.bmj.com/site/about/guidelines.xhtml

Disorders Work Group. Int J Geriatr Psychiatry. 2012;27(6):549-56. doi:10.1002/gps.2752.

23 Collins RN, Kishita N. Prevalence of depression and burden among informal care-givers of people with dementia: a meta-analysis. Aging Soc. 2020;40(11):2355-92. https://doi.org/10.1017/S0144686X19000527.

24 Contreras ML, Mioshi E, Kishita N. Factors related to the quality of life in family carers of people with dementia: a meta-analysis. J Geriatr Psychiatry Neurol. 2021;34(5):482-500. doi:10.1177/0891988720924713.

25 Ferraris G, Fisher O, Lamura G, et al. Dyadic associations between perceived social support and psychological well-being in caregivers and older care recipients. J Fam Psychol. 2022;36(8):1397-1406. doi:10.1037/fam0001009.

26 Dechartres A, Trinquart L, Faber T, et al. Empirical evaluation of which trial characteristics are associated with treatment effect estimates. J Clin Epidemiol. 2016;77:24-37. doi:10.1016/j.jclinepi.2016.04.005.

27 Saltaji H, Armijo-Olivo S, Cummings GG, et al. Impact of Selection Bias on Treatment Effect Size Estimates in Randomized Trials of Oral Health Interventions: A Meta-epidemiological Study. J Dent Res. 2018;97(1):5-13. doi:10.1177/0022034517725049.

28 Collins KA, Westra HA, Dozois DJ, et al. Gaps in accessing treatment for anxiety and depression: challenges for the delivery of care. Clin Psychol Rev. 2004;24(5):583-616. doi:10.1016/j.cpr.2004.06.001.

29 Kenning C, Daker-White G, Blakemore A, et al. Barriers and facilitators in accessing dementia care by ethnic minority groups: a meta-synthesis of qualitative studies. BMC Psychiatry. 2017;17(1):316. doi:10.1186/s12888-017-1474-0.

30 Stephan A, Bieber A, Hopper L, et al. Barriers and facilitators to the access to and use of formal dementia care: findings of a focus group study with people with dementia, informal carers and health and social care professionals in eight European countries. BMC Geriatr. 2018;18(1):131. doi:10.1186/s12877-018-0816-1.

31 Sekhon M, Cartwright M, Francis JJ. Acceptability of healthcare interventions: an overview of reviews and development of a theoretical framework. BMC Health Serv Res. 2017;17(1):88. doi:10.1186/s12913-017-2031-8.

32 Kruse C, Fohn J, Wilson N, et al. Utilization barriers and medical outcomes commensurate with the use of telehealth among older adults: systematic review. JMIR Med Inform. 2020;8(8):e20359. doi:10.2196/20359.

For peer review only - http://bmjopen.bmj.com/site/about/guidelines.xhtml

33 Bartlett YK, Kenning C, Crosland J, et al. Understanding acceptability in the context of text messages to encourage medication adherence in people with type 2 diabetes. BMC Health Serv Res. 2021;21(1):608. doi:10.1186/s12913-021-06663-2.

34 Hommel KA, Hente E, Herzer M, et al. Telehealth behavioral treatment for medication nonadherence: a pilot and feasibility study. Eur J Gastroenterol Hepatol. 2013;25(4):469-73. doi:10.1097/MEG.0b013e32835c2a1b.

35 Seckler E, Regauer V, Rotter T, et al. Barriers to and facilitators of the implementation of multi-disciplinary care pathways in primary care: a systematic review. BMC Fam Pract. 2020;21(1):113. doi:10.1186/s12875-020-01179-w.

36 Hoffmann TC, Glasziou PP, Boutron I, et al. Better reporting of interventions: template for intervention description and replication (TIDieR) checklist and guide. BMJ. 2014;348:g1687. doi:10.1136/bmj.g1687.

37 Vassar M, Pollard J, Rorah D, et al. Assessment of the completeness of intervention reporting of randomized clinical trials for alcohol use disorders: effect of the TIDieR checklist and guide. Drug Alcohol Depend. 2020;208:107824. doi:10.1016/j.drugalcdep.2019.107824.

38 Stevens A, Shamseer L, Weinstein E, et al. Relation of completeness of reporting of health research to journals' endorsement of reporting guidelines: a systematic review. BMJ. 2014;348:g3804. doi:10.1136/bmj.g3804.

39 Anderson JM, Stafford A, Jellison S, et al. Intervention reporting of published trials is insufficient in orthopedic surgery journals: application of the template for intervention description and replication checklist. Arthrosc Sports Med Rehabil. 2021;3(3):e619-27. doi:10.1016/j.asmr.2020.09.019.

40 Sterne JAC, Savović J, Page MJ, et al. RoB 2: a revised tool for assessing risk of bias in randomised trials. BMJ. 2019;366:l4898. doi:10.1136/bmj.l4898.

41 Moher D, Shamseer L, Clarke M, et al. Preferred reporting items for systematic review and meta-analysis protocols (PRISMA-P) 2015 statement. Syst Rev. 2015;4(1):1. doi:10.1186/2046-4053-4-1.

42 Tong A, Flemming K, McInnes E, et al. Enhancing transparency in reporting the synthesis of qualitative research: ENTREQ. BMC Med Res Methodol. 2012;12:181. doi:10.1186/1471-2288-12-181.

43 Page MJ, McKenzie JE, Bossuyt PM, et al. The PRISMA 2020 statement: an updated guideline for reporting systematic reviews. BMJ. 2021;372:n71. doi:10.1136/bmj.n71.

For peer review only

44 Farrand P, Matthews J, Dickens C, et al. Psychological interventions to improve psychological well-being in people with dementia or mild cognitive impairment: systematic review and meta-analysis protocol. BMJ Open. 2016;6(1):e009713. doi:10.1136/bmjopen-2015-009713.

45 Centre for Reviews and Dissemination (CRD). Systematic reviews: CRD's guidance for undertaking reviews in health care. York: Centre for Reviews and Dissemination, University of York; 2009.

46 Higgins JP, Eldridge S, Li T. Including variants on randomized trials. In: Higgins JP, Thomas J, Chandler J, Cumpston M, Li T, Page MJ, editors, et al. Cochrane handbook for systematic reviews of interventions. New Jersey: John Wiley & Sons; 2019. doi:10.1002/9781119536604.ch23.

47 Lizarondo L, Stern C, Carrier J, et al. Chapter 8: Mixed methods systematic reviews. In: Aromataris E, Munn Z (Editors). JBI Manual for Evidence Synthesis. Adelaide: JBI 2020. doi:10.46658/JBIMES-20-09.

48 Evans SR. Fundamentals of clinical trial design. J Exp Stroke Transl Med. 2010;3:19-27. doi:10.6030/1939-067x-3.1.19.

49 Mohr DC, Spring B, Freedland KE, et al. The selection and design of control conditions for randomized controlled trials of psychological interventions. Psychother Psychosom. 2009;78:275-84. doi:10.1159/000228248.

50 Viertiö S, Kiviruusu O, Piirtola M, et al. Factors contributing to psychological distress in the working population, with a special reference to gender difference. BMC Public Health. 2021;21(1):611. doi:10.1186/s12889-021-10560-y.

51 Milosevic I, Levy HC, Alcolado GM, et al. The Treatment Acceptability/Adherence Scale: moving beyond the assessment of treatment effectiveness. Cogn Behav Ther. 2015;44(6):456-69. doi:10.1080/16506073.2015.1053407.

52 Farrand P, Woodford J. Impact of support on the effectiveness of written cognitive behavioural self-help: a systematic review and meta-analysis of randomised controlled trials. Clin Psychol Rev. 2013;33(1):182-95. doi:10.1016/j.cpr.2012.11.001.

53 Farrand P, Woodford J. Effectiveness of cognitive behavioural self-help for the treatment of depression and anxiety in people with long-term physical health conditions: a systematic review and meta-analysis of randomised controlled trials. Ann Behav Med. 2015;49(4):579-93. doi:10.1007/s12160-015-9689-0.

54 Mårtensson E, Blomberg O, Pettman D, et al. Psychological interventions for depression among informal caregivers of older adult populations: protocol of a

For peer review only - http://bmjopen.bmj.com/site/about/guidelines.xhtml

systematic review and meta-analysis of randomised controlled trials. BMJ Open. 2020;10(9):e036402. doi:10.1136/bmjopen-2019-036402.

55 Pettman D, O'Mahen H, Skoog Svanberg A, et al. Effectiveness and acceptability of cognitive-behavioural therapy based interventions for maternal peripartum depression: a systematic review, meta-analysis and thematic synthesis protocol. BMJ Open. 2019;9(12):e032659. doi:10.1136/bmjopen-2019-032659.

56 Bramer WM, Giustini D, de Jonge GB, et al. De-duplication of database search results for systematic reviews in EndNote [published correction appears in J Med Libr Assoc. 2017 Jan;105(1):111]. J Med Libr Assoc. 2016;104(3):240-3. doi:10.3163/1536-5050.104.3.014.

57 Ouzzani M, Hammady H, Fedorowicz Z, et al. Rayyan-a web and mobile app for systematic reviews. Syst Rev. 2016;5(1):210. doi:10.1186/s13643-016-0384-4.

58 QSR International Pty Ltd. NVivo qualitative data analysis software, Version 12. QSR International Pty Ltd, Victoria. 2021. http://www.qsrinternational.com/nvivo. Accessed 5 May 2023.

59 McGuinness LA, Higgins JPT. Risk-of-bias VISualization (robvis): An R package and shiny web app for visualizing risk-of-bias assessments. Res Synth Methods. 2021;12(1):55-61. doi:10.1002/jrsm.1411.

60 Schünemann HJ, Oxman AD, Brozek J, et al. Grading quality of evidence and strength of recommendations for diagnostic tests and strategies [published correction appears in BMJ. 2008 May 24;336(7654). doi:10.1136/bmj.a139. Schünemann, A Holger J [corrected to Schünemann, Holger J]]. BMJ. 2008;336(7653):1106-10. doi:10.1136/bmj.39500.677199.AE.

61 Guyatt GH, Oxman AD, Vist GE, et al. GRADE: an emerging consensus on rating quality of evidence and strength of recommendations. BMJ. 2008;336(7650):924-6. doi:10.1136/bmj.39489.470347.AD.

62 Ryan R, Hill S. How to grade the quality of the evidence. Cochrane Consum Commun Gr. 2016:1-25.

63 Critical Appraisal Skills Programme (CASP). CASP qualitative checklist. CASP. 2017. https://casp-uk.net/images/checklist/documents/CASP-Qualitative-Studies-Checklist/CASP-Qualitative-Checklist-2018_fillable_form.pdf. Accessed 5 May 2023.

64 Hedges V. Distribution theory for Glass's estimator of effect size and related estimators. J Educ Behav Stat. 1981;6(2):107-28. doi:10.2307/1164588.

For peer review only - http://bmjopen.bmj.com/site/about/guidelines.xhtml

65 Ekers D, Webster L, Van Straten A, et al. Behavioural activation for depression; an update of meta-analysis of effectiveness and sub group analysis. PLoS One. 2014;9(6):e100100. doi:10.1371/journal.pone.0100100.

66 Flückiger C, Del Re AC, Munder T, et al. Enduring effects of evidence-based psychotherapies in acute depression and anxiety disorders versus treatment as usual at follow-up - a longitudinal meta-analysis. Clin Psychol Rev. 2014;34(5):367-75. doi:10.1016/j.cpr.2014.05.001.

67 Simblett S, Birch J, Matcham F, et al. A systematic review and meta-analysis of e-mental health interventions to treat symptoms of posttraumatic stress. JMIR Ment Health. 2017;4(2):e14. doi:10.2196/mental.5558.

68 Schmidt FL, Oh IS, Hayes TL. Fixed- versus random-effects models in meta-analysis: model properties and an empirical comparison of differences in results. Br J Math Stat Psychol. 2009;62(Pt1):97-128. doi:10.1348/000711007X255327.

69 Higgins JP, Thompson SG. Quantifying heterogeneity in a meta-analysis. Stat Med. 2002;21(11):1539-58. doi:10.1002/sim.1186.

70 Borenstein M. In a meta-analysis, the I-squared statistic does not tell us how much the effect size varies. J Clin Epidemiol. 2022;152:281-4. doi:10.1016/j.jclinepi.2022.10.003.

71 Higgins JP, Thompson SG, Deeks JJ, et al. Measuring inconsistency in meta-analyses. BMJ. 2003;327(7414):557-60. doi:10.1136/bmj.327.7414.557.

72 Duval S, Tweedie R. Trim and fill: a simple funnel-plot-based method of testing and adjusting for publication bias in meta-analysis. Biometrics. 2000;56(2):455-63. doi:10.1111/j.0006-341x.2000.00455.x.

73 Bartlett YK, Kenning C, Crosland J, et al. Understanding acceptability in the context of text messages to encourage medication adherence in people with type 2 diabetes. BMC Health Serv Res. 2021;21(1):608. doi:10.1186/s12913-021-06663-2.

74 Paskins Z, Crawford-Manning F, Cottrell E, et al. Acceptability of bisphosphonates among patients, clinicians and managers: a systematic review and framework synthesis. BMJ Open. 2020;10(11):e040634. doi:10.1136/bmjopen-2020-040634.

75 Murphy AL, Gardner DM. Pharmacists' acceptability of a men's mental health promotion program using the Theoretical Framework of Acceptability. AIMS Public Health. 2019;6(2):195-208. doi:10.3934/publichealth.2019.2.195.

76 Creswell JW, Miller DL. Determining validity in qualitative inquiry. Theory into practice. 2000;39(3):124-30. doi:10.1207/s15430421tip3903_2.

For peer review only - http://bmjopen.bmj.com/site/about/guidelines.xhtml

77 Yamato TP, Maher CG, Saragiotto BT, et al. Rasch analysis suggested that items from the template for intervention description and replication (TIDieR) checklist can be summed to create a score. J Clin Epidemiol. 2018;101:28-34. doi:10.1016/j.jclinepi.2018.05.014.

78 Pollock A, Campbell P, Struthers C, et al. Stakeholder involvement in systematic reviews: a scoping review. Syst Rev. 2018;7(1):208. doi:10.1186/s13643-018-0852-0.

79 Ma LL, Wang YY, Yang ZH, et al. Methodological quality (risk of bias) assessment tools for primary and secondary medical studies: what are they and which is better? Mil Med Res. 2020;7(1):7. doi:10.1186/s40779-020-00238-8.

80 Morrison A, Polisena J, Husereau D, et al. The effect of English-language restriction on systematic review-based meta-analyses: a systematic review of empirical studies. Int J Technol Assess Health Care. 2012;28(2):138-44. doi:10.1017/S0266462312000086.

81 Skivington K, Matthews L, Simpson SA, et al. A new framework for developing and evaluating complex interventions: update of Medical Research Council guidance. BMJ. 2021;374:n2061. doi:10.1136/bmj.n2061.

82 Svedin F, Brantnell A, Farrand P, et al. Adapting a guided low-intensity behavioural activation intervention for people with dementia and depression in the Swedish healthcare context (INVOLVERA): a study protocol using codesign and participatory action research. BMJ Open. 2021;11(7):e048097. doi:10.1136/bmjopen-2020-048097.

For peer review only

PRISMA-P (Preferred Reporting Items for Systematic review and Meta-Analysis Protocols) 2015 checklist: recommended items to address in a systematic review protocol*

Section and topic	Item No	Checklist item	Reported on Page
**ADMINISTRATIVE INFORMATION**			
Title:			
Identification	1a	Identify the report as a protocol of a systematic review	Page 0
Update	1b	If the protocol is for an update of a previous systematic review, identify as such	N/A
Registration	2	If registered, provide the name of the registry (such as PROSPERO) and registration number	Page 1
Authors:			
Contact	3a	Provide name, institutional affiliation, e-mail address of all protocol authors; provide physical mailing address of corresponding author	Page 0
Contributions	3b	Describe contributions of protocol authors and identify the	Page 19
Amendments	4	If the protocol represents an amendment of a previously completed or published protocol, identify as such and list changes; otherwise, state plan for documenting important protocol amendments	N/A
Support:			
Sources	5a	Indicate sources of financial or other support for the review	Page 19
Sponsor	5b	Provide name for the review funder and/or sponsor	Page 19
Role of sponsor or funder	5c	Describe roles of funder(s), sponsor(s), and/or institution(s), if any, in developing the protocol	N/A
**INTRODUCTION**			
Rationale	6	Describe the rationale for the review in the context of what is already known	Page 3-5
Objectives	7	Provide an explicit statement of the question(s) the review will address with reference to participants, interventions, comparators, and outcomes (PICO)	Page 5-6

For peer review only

**METHODS**			
Eligibility criteria	8	Specify the study characteristics (such as PICO, study design, setting, time frame) and report characteristics (such as years considered, language, publication status) to be used as criteria for eligibility for the review	Page 6-8
Information sources	9	Describe all intended information sources (such as electronic databases, contact with study authors, trial registers or other grey literature sources) with planned dates of coverage	Page 8-9
Search strategy	10	Present draft of search strategy to be used for at least one electronic database, including planned limits, such that it could be repeated	Appendix 2
Study records:			
Data management	11a	Describe the mechanism(s) that will be used to manage records and data throughout the review	Page 9
Selection process	11b	State the process that will be used for selecting studies (such as two independent reviewers) through each phase of the review (that is, screening, eligibility and inclusion in meta-analysis)	Page 9-11
Data collection process	11c	Describe planned method of extracting data from reports (such as piloting forms, done independently, in duplicate), any processes for obtaining and confirming data from investigators	Page 9-11
Data items	12	List and define all variables for which data will be sought (such as PICO items, funding sources), any pre-planned data assumptions and simplifications	Page 9-11
Outcomes and prioritization	13	List and define all outcomes for which data will be sought, including prioritization of main and additional outcomes, with rationale	Page 7-8, 12-13
Risk of bias in individual studies	14	Describe anticipated methods for assessing risk of bias of individual studies, including whether this will be done at the outcome or study level, or both; state how this information will be used in data synthesis	Page 11-12
Data synthesis	15a	Describe criteria under which study data will be quantitatively synthesised	Page 12-15
	15b	If data are appropriate for quantitative synthesis, describe planned summary measures, methods of handling data and methods of combining data from studies, including any planned exploration of consistency (such as I^2, Kendall's τ)	Page 12-16

For peer review only

	15c	Describe any proposed additional analyses (such as sensitivity or subgroup analyses, meta-regression)	Page 14
	15d	If quantitative synthesis is not appropriate, describe the type of summary planned	Page 12-13
Meta-bias(es)	16	Specify any planned assessment of meta-bias(es) (such as publication bias across studies, selective reporting within studies)	Page 14
Confidence in cumulative evidence	17	Describe how the strength of the body of evidence will be assessed (such as GRADE)	Page 12

*** It is strongly recommended that this checklist be read in conjunction with the PRISMA-P Explanation and Elaboration (cite when available) for important clarification on the items. Amendments to a review protocol should be tracked and dated.** **The copyright for PRISMA-P (including checklist) is held by the PRISMA-P Group and is distributed under a Creative Commons Attribution Licence 4.0.**

From: Shamseer L, Moher D, Clarke M, Ghersi D, Liberati A, Petticrew M, Shekelle P, Stewart L, PRISMA-P Group. Preferred reporting items for systematic review and meta-analysis protocols (PRISMA-P) 2015: elaboration and explanation. BMJ. 2015 Jan 2;349(jan02 1):g7647.

For peer review only

Enhancing transparency in reporting the synthesis of qualitative research: ENTREQ Checklist (Tong, et al., 2012)

Item No.	Guide and Description	Reported on Page
1. Aim	State the research question the synthesis addresses	5-6
2. Synthesis methodology	Identify the synthesis methodology or theoretical framework which underpins the synthesis, and describe the rationale for choice of methodology (e.g. meta-ethnography, thematic synthesis, critical interpretive synthesis, grounded theory synthesis, realist synthesis, meta-aggregation, meta-study, framework synthesis)	14-15
3. Approach to searching	Indicate whether the search was pre-planned (comprehensive search strategies to seek all available studies) or iterative (to seek all available concepts until they theoretical saturation is achieved)	8
4. Inclusion criteria	Specify the inclusion/exclusion criteria (e.g. in terms of population, language, year limits, type of publication, study type)	6-8
5. Data sources	Describe the information sources used (e.g. electronic databases (MEDLINE, EMBASE, CINAHL, psycINFO), grey literature databases (digital thesis, policy reports), relevant organisational websites, experts, information specialists, generic web searches (Google Scholar) hand searching, reference lists) and when the searches conducted; provide the rationale for using the data sources	8-9
6. Electronic Search strategy	Describe the literature search (e.g. provide electronic search strategies with population terms, clinical or health topic terms, experiential or social phenomena related terms, filters for qualitative research, and search limits)	8-9
7. Study screening methods	Describe the process of study screening and sifting (e.g. title, abstract and full text review, number of independent reviewers who screened studies)	9
8. Study characteristics	Present the characteristics of the included studies (e.g. year of publication, country, population, number of participants, data collection, methodology, analysis, research questions)	N/A
9. Study selection results	Identify the number of studies screened and provide reasons for study exclusion (e.g. for comprehensive searching, provide numbers of studies screened and reasons for exclusion indicated in a figure/flowchart; for iterative searching describe reasons for study exclusion and inclusion based on modifications to the research question and/or contribution to theory development)	N/A

For peer review only

10. Rationale for appraisal	Describe the rationale and approach used to appraise the included studies or selected findings (e.g. assessment of conduct (validity and robustness), assessment of reporting (transparency), assessment of content and utility of the findings)	N/A
11. Appraisal items	State the tools, frameworks and criteria used to appraise the studies or selected findings (e.g. Existing tools: CASP, QARI, COREQ, Mays and Pope [25]; reviewer developed tools; describe the domains assessed: research team, study design, data analysis and interpretations, reporting)	12
12. Appraisal process	Indicate whether the appraisal was conducted independently by more than one reviewer and if consensus was required	12
13. Appraisal results	Present results of the quality assessment and indicate which articles, if any, were weighted/excluded based on the assessment and give the rationale	N/A
14. Data extraction	Indicate which sections of the primary studies were analysed and how were the data extracted from the primary studies? (e.g. all text under the headings "results /conclusions" were extracted electronically and entered into a computer software)	11
15. Software	State the computer software used, if any	11
16. Number of reviewers	Identify who was involved in coding and analysis	14-15
17. Coding	Describe the process for coding of data (e.g. line by line coding to search for concepts)	14-15
18. Study comparison	Describe how were comparisons made within and across studies (e.g. subsequent studies were coded into pre-existing concepts, and new concepts were created when deemed necessary)	N/A
19. Derivation of themes	Explain whether the process of deriving the themes or constructs was inductive or deductive	14-15
20. Quotations	Provide quotations from the primary studies to illustrate themes/constructs, and identify whether the quotations were participant quotations of the author's interpretation	N/A
21. Synthesis output	Present rich, compelling and useful results that go beyond a summary of the primary studies (e.g. new interpretation, models of evidence, conceptual models, analytical framework, development of a new theory or construct)	N/A

For peer review only

Database: *Ovid MEDLINE*

Search nr	Term	Comment
Concept 1 – Population		
1	exp Dementia/<br>OR exp Cognition Disorders/<br>OR Cognitive Dysfunction/	[MeSH]
2	(dement* or Alzheimer* or lewy* or frontotemporal or FTD or FTLD or tvFTD or OBS or OBD or (lewy* adj2 bod*) or organic brain syndrome* or organic brain disease or organic brain disorder* or (ADRD or AAMI or AACD or MCI or A-MCI or N-MCI or M-MCI or aMCI or MCIa or CIND or MCD or MNC or MNCD or NCD) or ag* associated cogniti* decline or ag* associated memory impairment or mild cognitive impairment or neurocognitive disorder* or preclinical AD or pre-clinical AD or (pre adj clinical AD)).ti,ab.	[Title/Abstract]
3	1 OR 2	
Concept 2 – Intervention		
4	exp Counseling/<br>OR exp Behavior Therapy/<br>OR exp Psychotherapy/<br>OR exp Bibliotherapy/	[MeSH]
5	((non adj pharmacologic*) or non-pharmacologic* or nonpharmacologic* or cognitive restructuring or cognitive reframing or behavio* activation or activity scheduling or problem solving or (cCBT or iCBT or ehealth or e-health or teletherapy or telehealth) or (self adj help) or (self adj manag*) or (self adj administer*) or (psycho* adj therapy) or (cognitive adj2 therap*) or (behavio* adj2 therap*) or (CBT or psychotherapy or psychodynamic or counseling or counselling or psychoeducation* or psychosocial or psycho-social) or (group adj therap*) or (group adj treatment*) or (group adj intervention*) or (group adj support) or psycho education*).ti,ab.	[Title/Abstract]
6	4 OR 5	
Concept 3 – Outcome		
7	exp Depression/<br>OR exp Anxiety/<br>OR exp Anxiety Disorders/<br>OR exp Mood Disorders/<br>OR Affective Symptoms/<br>OR Psychological Distress/	[MeSH]
8	("quality of life" or (mental adj health) or (mental adj distress) or (mental adj illness) or (psycholo*	[Title/Abstract]

For peer review only - http://bmjopen.bmj.com/site/about/guidelines.xhtml

	adj distress) or (neuropsycholog* or neuropsychiatric) or (mood or emotion* or affective or wellbeing or well-being or distress) or (negative adj affect) or Depress* or Melanchol* or Dysphori* or (anxiety or anxious or stress or worr*) or (well adj being)).ti,ab.	
9	7 OR 8	
Concept 4 – Study design		
10	exp Randomized Controlled Trial/<br>OR exp Clinical Trial/<br>OR Meta-Analysis/<br>OR Random Allocation/	[MeSH]
11	(random* controlled trial* or (RCT or Trial or review or meta-analysis) or (random* adj allocat*)).ti,ab.	[Title/Abstract]
12	10 OR 11	
Final results	3 AND 6 AND 9 AND 12	

For peer review only
